# Proteomic Analysis and qRT-PCR Verification of Temperature Response to *Arthrospira* (*Spirulina*) *platensis*

**DOI:** 10.1371/journal.pone.0083485

**Published:** 2013-12-12

**Authors:** Wang Huili, Zhao Xiaokai, Lin Meili, Randy A. Dahlgren, Chen Wei, Zhou Jaiopeng, Xu Chengyang, Jin Chunlei, Xu Yi, Wang Xuedong, Ding Li, Bao Qiyu

**Affiliations:** 1 Institute of Biomedical Informatics/Zhejiang Provincial Key Laboratory of Medical Genetics, School of Life Sciences, Wenzhou Medical University, Wenzhou, China; 2 Department of Land, Air and Water Resources, University of California Davis, Davis, California, United States of America; 3 School of Environmental Sciences and Public Health, Wenzhou Medical University, Wenzhou, China; Lawrence Berkeley National Laboratory, United States of America

## Abstract

*Arthrospira* (*Spirulina*) *platensis* (ASP) is a representative filamentous, non-N_2_-fixing cyanobacterium that has great potential to enhance the food supply and possesses several valuable physiological features. ASP tolerates high and low temperatures along with highly alkaline and salty environments, and can strongly resist oxidation and irradiation. Based on genomic sequencing of ASP, we compared the protein expression profiles of this organism under different temperature conditions (15°C, 35°Cand 45°C) using 2-DE and peptide mass fingerprinting techniques. A total of 122 proteins having a significant differential expression response to temperature were retrieved. Of the positively expressed proteins, the homologies of 116 ASP proteins were found in *Arthrospira* (81 proteins in *Arthrospira platensis* str. Paraca and 35 in *Arthrospira maxima* CS-328). The other 6 proteins have high homology with other microorganisms. We classified the 122 differentially expressed positive proteins into 14 functions using the COG database, and characterized their respective KEGG metabolism pathways. The results demonstrated that these differentially expressed proteins are mainly involved in post-translational modification (protein turnover, chaperones), energy metabolism (photosynthesis, respiratory electron transport), translation (ribosomal structure and biogenesis) and carbohydrate transport and metabolism. Others proteins were related to amino acid transport and metabolism, cell envelope biogenesis, coenzyme metabolism and signal transduction mechanisms. Results implied that these proteins can perform predictable roles in rendering ASP resistance against low and high temperatures. Subsequently, we determined the transcription level of 38 genes *in vivo* in response to temperature and identified them by qRT-PCR. We found that the 26 differentially expressed proteins, representing 68.4% of the total target genes, maintained consistency between transcription and translation levels. The remaining 12 genes showed inconsistent protein expression with transcription level and accounted for 31.6% of the total target genes.

## Introduction

*Arthrospira* (*Spirulina*) *platensis* (ASP) is a genera of filamentous, non-heterocyst-forming cyanobacteria. It is gram-negative, has no flagellum and contains chlorophyll-a that carries out photosynthesis producing oxygen. It is well known for its useful application as a food source [1]. ASP possesses abundant proteins representing 60-70% of its dry weight and its protein composition is nearly perfect for human nutrition. It contains eight necessary amino acids, various unsaturated fatty acids, vitamins, mineral substances and biologically active agents which are necessary for the human body. These substances provide increased immunity, anti-cancer and anti-aging benefits, radiation resistance, antioxidant properties, and reduction to drug toxicity effects. Consequently, ASP is widely applied to several fields, such as functional food, compound feedstuff, new model medicines and fine chemicals [2]. 

Scientists have found that this organism also possesses some unusual but valuable physiological features, such as adaptation to highly alkaline environments, resistance to high or low temperatures, antioxidant activity, and radiation and salt tolerance. Due to these properties, it often occupies a dominant ecological position in lakes with high carbonate/bicarbonate levels. One of the most common stresses for many living organisms is extreme variation in their surrounding temperature. Adaptive mechanisms that protect against the potentially harmful effects of elevated or reduced temperatures are found in all living organisms. These survival pathways are primarily regulated at the transcription level and then at post-transcription and translation levels [3].

In recent years, heat-shock or cold-shock response in some cyanobacteria, such as *Synechocystis*, *Synechococcus* and *Nostoc*, has been investigated [4–6]. Kurdrid et al. [7] demonstrated that the heat-shock protein (Hsp) and an alternative sigma factor (SigH) in *Spirulina plantensis* were significantly induced immediately after exposure to high temperature. Moreover, expression of the HspA gene in *Synechococcus* was controlled via the binding of regulatory proteins to the upstream AT-rich region [8]. Immunocytochemical studies by Kojima showed that the main localization of HspA in the cyanobacterium *Synechococcus* strain ECT16-1 cells shifted from the thylakoid area to the cytoplasm, then back to the thylakoid area during heat stress [8]. Expression of HspA stabilizes the morphology of nucleoids and this unique property of HspA is associated with thylakoid membranes [7]. Balogi et al. [9] observed an increase in the level of the highly saturated monoglucosyldiacylglycerol (MGlcDG) in *Synechocystis* cells. The MGlcDG membranes remain stable even at extremely high temperatures (45°C) and express the strongest interaction with the thylakoid-stabilizing Hsp17 from *Synechocystis*. They suggest that the highly saturated MGlcDG functions as a “heat-shock lipid” and is of potential importance in the development of acquired thermotolerance of heat/light-primed cyanobacterial thylakoids [9]. Similarly, the desD gene that encodes Δ6-desaturase of *Spirulina platensis* is regulated at the transcription level in response to temperature reduction. This regulation occurs via a protein complex, consisting of the GntR regulatory protein and the GroEL chaperone [10]. The effects of low and high temperatures on the transcription of these desaturase genes in *Spirulina* have been previously reported. A temperature change from 35 to 40°C in the presence of light does not significantly affect either the mRNA levels of desC, desA and desD genes or the stability of the desD mRNA [11].

At present, due to the lack of whole-genome sequences for ASP, proteomics research examining environmental stress is limited. As a result, proteome studies of ASP are still based on sub-cell levels, such as the thylakoid membrane, plasma membrane, etc. [12]. In addition, much of the previous research has focused on the search for specific genes or regulating factors, while little data are available on gene functional research using a systematic approach [13,14,15]. This paper aimed to analyze the protein expression of ASP using 2-dimensional gel electrophoresis (2-DE) and MALDI-TOF/MS to further explore temperature response, track the differential expression spectrum, identify the differentially expressed proteins, and analyze their main physiological functional classification. On this basis, we discussed the resistance mechanisms of ASP under different temperature conditions. Additionally, we used qRT-PCR to validate the consistency of subset genes between transcription and translation levels. This research can inform a better understanding of environmental adaptation mechanisms of cyanobacteria, and provides basic clues to further develop ASP’s gene resources, which have the ability to tolerate several adverse environmental conditions.

## Material and Methods

### Culture conditions and sample preparation

ASP-YZ supplied by the Chinese Academy of Agricultural Sciences (Beijing, China) was cultured in 0.02 M NaCl Zarrouk medium at 35°C, 8 klx intensity and 75% humidity [16]. At the logarithmic growth period, ASP-YZ cells were transferred into fresh Zarrouk medium (initial inoculated concentration of OD_560_=0.2) at three different temperatures (15°C for low temperature, 35°C for control and 45°C for high temperature). Three biological replicates were established for each temperature treatment, i.e., three conical flasks of ASP (500 mL/flask) were cultured at each temperature (15, 35 or 45°C) for 7 d. Then, we collected the filaments from each flask. The harvested cells were washed three times with phosphate-based buffer, and then centrifuged at 8,000 rpm for 5 min to pellet old and dead cells. The cells in the supernatant were washed five times with ultra-pure water to ensure complete removal of salt. Finally, 9 individual filamentous samples were obtained, and stored at -80°C for further protein extraction.

### Protein extraction

First, the 9 individual filamentous samples prepared as described above were immediately frozen in liquid nitrogen for 10-15 min. Then, they were sedimented using trichloroacetic acid (TCA)/acetone as described in Giaralisco et al. [17], which resulted in protease inactivation, inhibition of protein degradation, and removal of impurities and extremely alkaline proteins. This TCA/acetone sediment technique is a routine method for extracting protein from cyanobacteria. After centrifugation, the precipitate was ultrasonically treated for 12 min in ice and resuspended with acetone, containing 0.07% BME at -20°C for 1-2 h, to fully remove pigments, then centrifuged at 35,000 g and 4°C for 15 min, and finally vacuum-dried. The protein pellets were then dissolved in an improved dissolving buffer, which contained 2 M thiourea, 5 M urea, 40 mM Tris, 20 mM DTT, 5 mM TCEP, 1% IPG buffer pH 3-10 (GE-Healthcare Biosciences, USA), 0.05% β-dodecyl maltoside and 2% CHAPS. The cell debris was discarded following centrifugation at 35,000 g for 30 min at 4°C. For expediency with subsequent experiments, the extracted proteins from the three biological replicates of each temperature treatment were composited into one sample. As a result, the 9 individual filamentous samples originating from the three temperature treatments yielded three mixed protein samples. The mixed protein concentration of the composited samples was determined using the DC-kit protein assay (Bio-Rad, USA) [7]. Finally, these protein samples were aliquoted, immediately frozen in liquid nitrogen and stored at -30 °C for later use.

### Two-dimensional gel electrophoresis

For one-DE, three technical replicates were established for each composited protein sample. The protein extract (22 µL) was diluted to a final concentration of 1000 µg/ml with an IEF rehydration solution, consisting of 17.5 µL DTT, 1.75 µL IPG (immobilized pH gradient buffer solution) and 309 µL R-Buffer (2 M sulfourea, 7 M urea, 4% CHAPS, 40 mM Tris, 2 mM TBP reductant and 0.2% Biolyte). Then, the above diluted protein extract (350 µL) was subjected to Immobiline DryStrips (pH 4-7; Length 17 cm; Amersham Bioscience). In a preliminary experiment, the protein isoelectric points (pI) for most *Spirulina* species were found to be pH 4-7 by means of drystrips in the pH range of 3-10. Therefore, the maximum separation was obtained by using the pH 4-7 drystrips. For the first-dimensional IEF (isoelectric focusing) based on the pI value of each protein, the procedures were performed as described by Blum et al. [18]. After completing the above IEF, 9 drystrips were used for electrophoresis in parallel using 12% polyacrylamid to produce 9 gels. The proteins on the gels were stained with the silver-based color development system using Gelcode (The Upjohn Co.). In order to improve the dynamic range of protein quantification in silver-stained gel and make it possible to visualize both high- and low-abundance proteins on a 2-DE gel, pre-processing of images was performed as described in Grove et al. [19]. Gels were fixed overnight in 50% ethanol/5% acetic acid, and rinsed (5 gels/tray) four times (1 h per rinse) in 1,800 ml of ultra-pure water. Following washing, the gels were equilibrated in 1.9 g/L AgNO_3_ (1 L/tray) for 60-90 min. An additional 1-h wash in Na_2_CO_3_ (7.5 g/L) followed the reducing step [7]. The protein spots were scanned using a UNAX Powerlook 2100XL (Bio-Rad) and analyzed by ImageMaster 2D platinum 5.0 (Amersham Bioscience) according to manufacturer’s instructions.

### Image acquisition and data analysis

Images were cropped and optimized before performing the inter-gel matching of the standard protein maps. Before spot matching, the internal standard image gel with the greatest number of spots was used as a master gel. The spot detection parameters were optimized by checking different protein spots in certain regions of the gel, which were then automatically detected, followed by visual inspection for removal or addition of undetected spots. The TwoClassDif (RVM-T test) method with a cutoff p-value <0.05 and FDR <0.05 was applied to analyze the differentially expressed proteins in the three different temperature treatments. Spot detection was refined by a manual spot edition where required. The spots that were visible on at least two gels from each temperature treatment or control based on the image analysis were identified as stably expressed protein spots. The abundance of each protein spot was estimated by the percentage volume (Vol%), that is, the spot volumes were normalized as a percentage of the total protein volume in all spots visible in the gel to correct for variability due to loading, gel staining and de-staining [20,21]. The percentage volumes were used to designate significant differential expression spots (at least three-fold increase/decrease and statistical significance as calculated by the RVM-T test with a cutoff *p*-value <0.05 and FDR <0.05). Triplicate gels were used for each sample. Only those with reproducible and significant changes were considered to be differentially expressed protein spots. Data were reported as mean ± standard deviation (mean ± SD), which was calculated by SPSS 16.0 software (SPSS, Chicago, IL, USA). The solely visible or not visible protein spots, scanned by UNAX Powerlook 2100XL, were judged by image analysis in a compared experimental group, such as 15°C vs 35°C, 45°C vs 35°C or 15°C vs 45°C. The significant differentially expressed protein spots were judged using the criterion >3-fold up-regulation or down-regulation.

### Protein identification by peptide mass fingerprinting (PMF) and MALDI-TOF MS analysis

The differentially expressed protein spots were manually selected and excised, then de-stained in 50% ACN in 25 mmol/L NH_4_HCO_3_ at 37°C for 30 min. Next, 50 µL ultra-pure H_2_O and 50 µL 50% ACN were added, followed by 100 µL of 100% ACN. The gels were rehydrated in 5 µL of trypsin (Promega, Madison, USA) solution (20 µg/ml in 25 mmol/L NH_4_HCO_3_) for 30 min. Next, 20 µL of cover solution (25 mmol/L NH_4_HCO_3_) was added, and digestion took place over night at 37°C. The supernatant was transferred into another tube, and the gels were extracted once with 50 µL extraction buffer (67% ACN and 5% TFA). The peptide extracts and the supernatant of the gel spot were combined and then completely dried. Samples were re-suspended in 5 µL 0.1% TFA followed by mixing in a 1:1 ratio with a matrix consisting of a saturated solution of CHCA in 50% ACN containing 0.1% TFA. The 1:1 mixture was spotted on a stainless steel sample target plate. The peptide samples were analyzed with a MALDI-TOF-MS Proteomics Analyzer (Bruker Daltonics, Bremen, Germany). The TOF spectra were recorded in positive ion reflector mode with a mass range from 800 to 4000 Da. About eight subspectra with 60 scans per subspectrum were accumulated to generate one main TOF spectrum. Data were searched on the Internet using a Mascot search engine (Matrix Science Ltd., London, UK) against all entries in the NCBInr database, which contained accessible public protein sequences (http://www.ncbi.nlm.nih.gov/nuccore/NZ_ACSK01001820.1/ *Arthrospira platensis* str. Paraca NZ_ACSK01001820 and http://www.ncbi.nlm.nih.gov/nuccore/NZ_ABYK00000000.1/ *Arthrospira* maxima CS-328), and the unpublished *Arthrospira* (*Spirulina*) database. The unpublished database was compiled by our group after nearly complete sequencing and annotation of *Arthrospira* (*Spirulina*) *platensis*-YZ originating from Beijing, China (The genome database of ASP-YZ is in the process of submission, but has not yet been published). All peptide masses were assumed monoisotopic and [M+H]^+^. The other parameters used for search were as follows: taxonomy, other bacteria; enzyme, trypsin; the fixed modification; carbamidomethyl (C); the variable modification, Glu- > pyro-Glu (N-term Q) and oxidation (M); mass toll = ±100 pm. The confidence in the peptide mass fingerprinting matches (*p* <0.05) was based on the MOWSE Score and confirmed by the accurate overlapping of the matched peptides with the major peaks of the mass spectrum. Only the significant hits, as defined by a MASCOT probability analysis (*p* <0.05), were accepted. If the theoretical values of MW and pI, which were identified by peptide mass fingerprinting (PMF) and MALDI-TOF/MS analysis, were consistent with the actual values analyzed by ImageMaster 2D platinum 5.0 in gel, we regarded this protein spot as a positive match. The matched peptides of this protein were blasted to the protein database nr (ftp://ftp.ncbi.nih.gov/blast/db/) and Swiss-Prot (ftp://ftp.uniprot.org/pub/databases/uniprot_datafiles_by_format/fasta/) in order to obtain the protein functional annotation information with the highest sequence similarity. Then, we acquired information on the ortholog of gene products, classification of gene functions and metabolic pathway analysis of gene products in cells by KEGG (ftp://ftp.genome.jp/pub/kegg/release/archive/kegg/) and COG (ftp://ftp.ncbi.nih.gov/pub/COG/) (evalue<0.00001) functional classification. These differentially expressed proteins were grouped by their respective metabolic pathways using the COG software online. In this investigation, our research group predicted temperature-responsive genes by metabolic pathway positioning through KEGG analysis, and selected part of the genes for further validation. Some of the selected genes comprised a large proportion of the total differentially expressed genes, and were possible temperature-responsive genes reported in previous reports [22,23,24], while the others were the up-regulated proteins or solely visible spots at 45°C or 15°C, while not visible at 35°C.

### qRT-PCR analyses of the differentially expressed genes

Primer sets, shown in Table S1, were designed for amplification of 16S rRNA and differentially expressed genes using Premier 5.0 software, and they were synthesized by Shanghai Sonny Biological Technology Co., Ltd. (Shanghai, China). To validate the differentially expressed proteins of ASP at the transcription level *in vivo*, qRT-PCR was performed using the StepOne^TM^ RT-PCR System (Applied Biosystem, USA). Three conical flasks of ASP were cultured for each temperature treatment to avoid loss due to pollution or error due to uneven temperatures. We collected the filaments and total RNA was extracted from the ASP-YZ cultured at control (35°C), low (15°C) and high (45°C) temperatures using a RNeasy Plant Mini Kit (Qiagen, Germany) according to manufacturer’s instructions and treated with Dnase I by RNase-Free Dnase Set (Qiagen, Germany). The extracted RNA for each temperature treatment was mixed into one sample, and thus a total of three RNA samples were obtained for the three temperature treatments. The quality of RNA was measured by the Nanodrop method and met the requirements: OD_260_/OD_280_ > 1.8 and OD_260_/OD _230_ > 1.5. After the RNA was detected without protein and salt contamination, cDNA was obtained by reverse transcription using a PrimeScript RT-PCR Kit (TaKaRa, Japan), following the procedure composed of an initial denaturation time of 5 min at 95°C, 35 cycles of amplification comprised of a denaturation step for 1 min at 95°C, and the annealing and extension temperatures as listed in Table S2. Each experimental group contained three technical replicates. At the same time, three PCR parallel tubes for control, containing Taq polymerase and extracted RNA as a template, were used to test for DNA contamination. Relative quantification of the targets in each sample was carried out using the signal of 16S rRNA as a stable reference gene [25].

 The copy numbers in two samples were first normalized, and the differentially expressed level was then calculated on the basis of the following equation:

$$\text{Folds}=\frac{G_{t}\times C_{h}}{C_{t}\times G_{h}}$$

Where G_t_, G_h_, C_t_ and C_h_ indicate the target gene of treatment, housekeeping gene of treatment, target gene of control and housekeeping gene of control, respectively.

## Results

### 2-DE profiles of total ASP-YZ proteins in response to different temperatures

**Figure 1:**
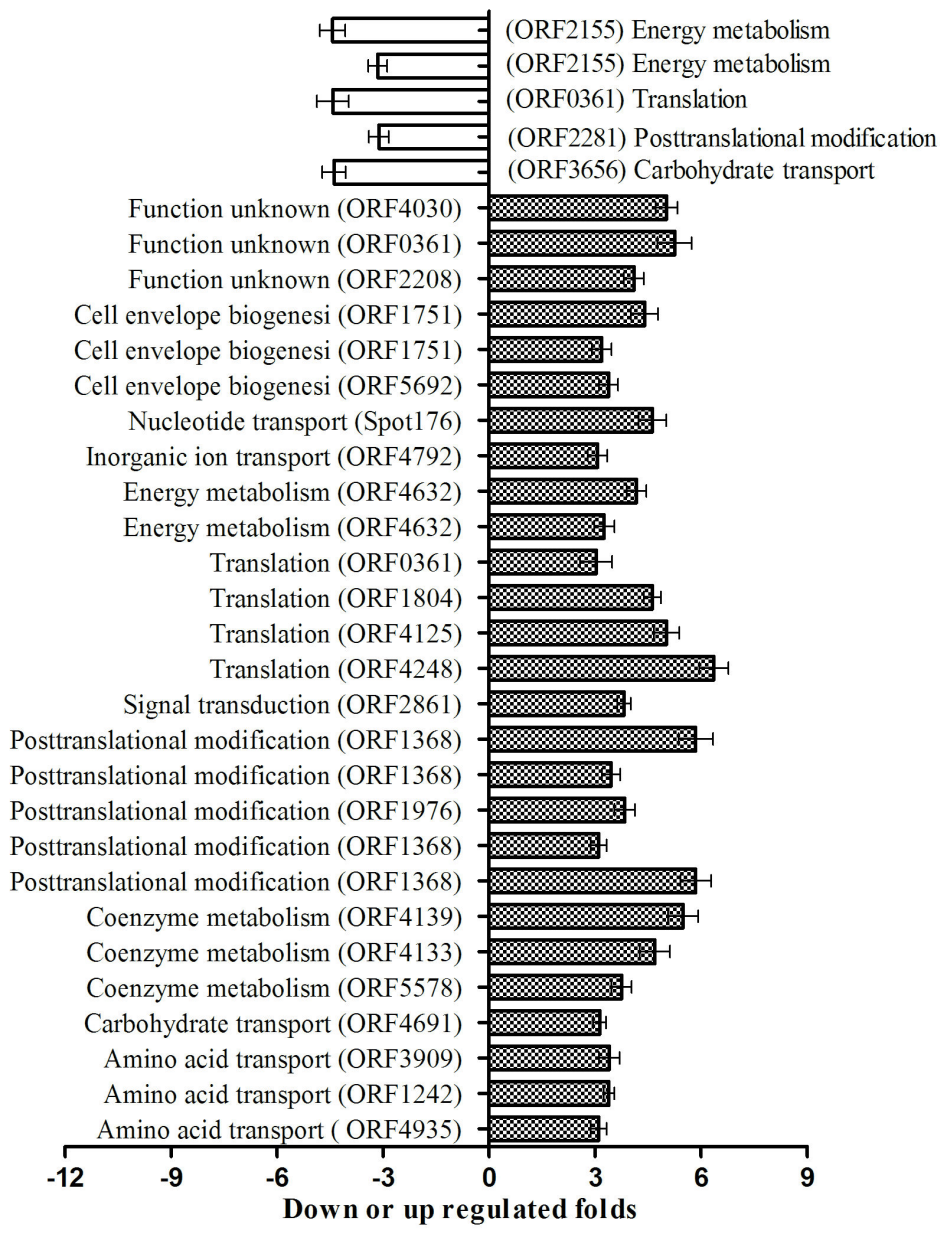
The differential protein spots between low temperature (15°C) treatment and the control (35°C). Note: The differential protein spots with >3.0-fold changes in low temperature (15°C) treatment compared with the control (35°C).

The differentially expressed protein profiles of ASP-YZ under three temperature conditions, control (35°C), low (15°C) and high (45°C), were analyzed by 2-DE with triplicate gels for each temperature group (see Figure 1A, 1B, 1C). The overall protein numbers of ASP-YZ were similar when cultured at different temperatures. There were 527 protein spots matched between the control (35°C) and 15°C gels (correlation coefficient (r) of 0.946 and matching rate of 83.7%), 468 between the control and 45°C gels (r = 0.954 and matching rate of 74.7%) and 489 between 15°C and 45°C gels (r = 0.964 and matching rate of 78.7%).

**Figure 2:**
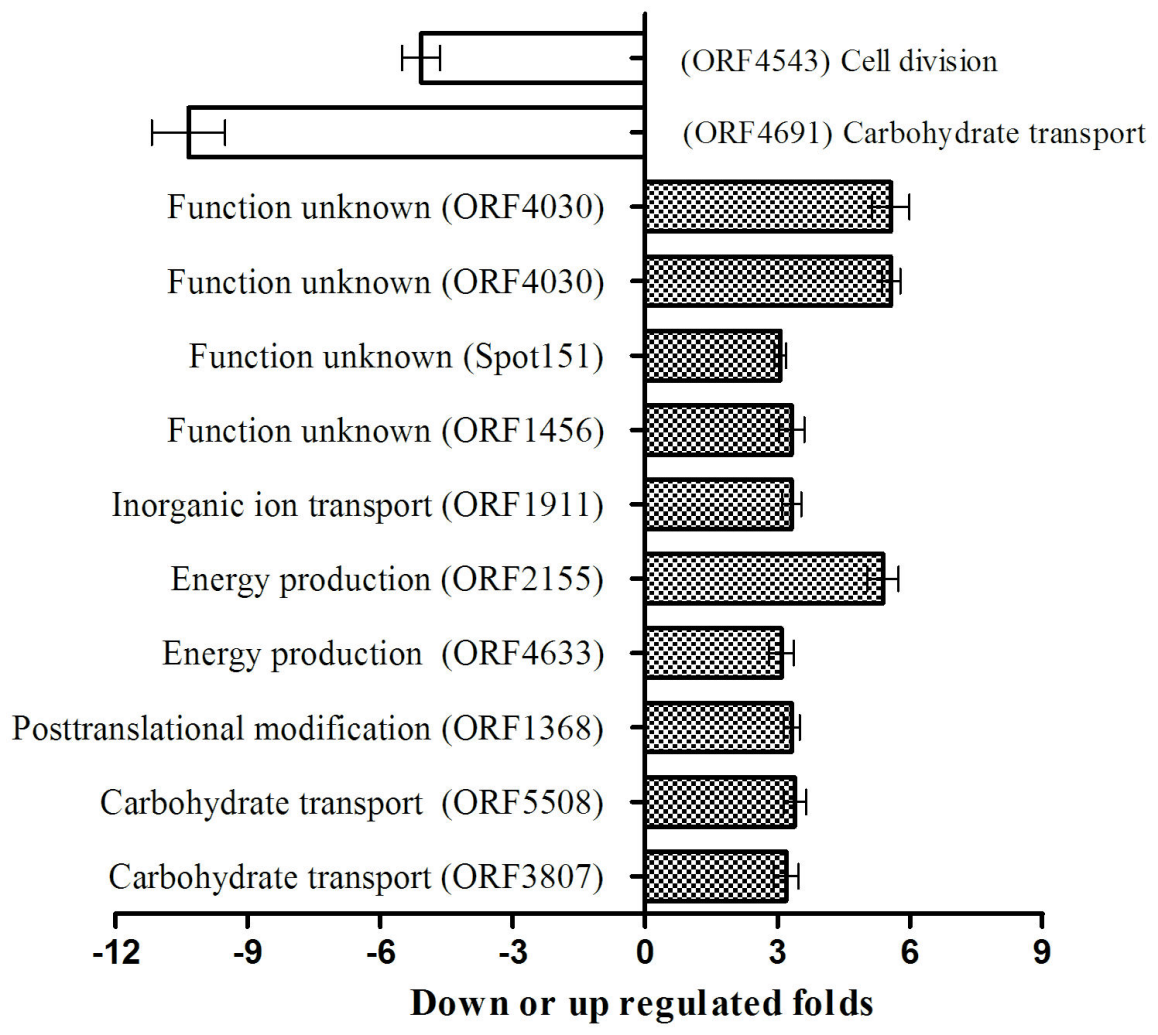
The differential protein spots between high temperature (45°C) treatment and control (35°C). Note: The differential protein spots with >3.0-fold changes in high temperature (45°C) treatment group compared to control (35°C).

Thirty-two protein spots and their fold changes were found to have more than a 3-fold higher differential expression compared to the control at 15°C (Figure 1). In addition, 18 proteins were solely visible at 15°C, while 21 proteins were solely visible for the control (Table 1). There were 12 protein spots which had over a 3-fold higher differential expression at 45°C compared to the control (Figure 2). In addition, 12 proteins were solely visible for the 45°C treatment, while 8 proteins were solely visible for the control (Table 2).

**Table 1:** The visible or not visible protein spots at 15°C vs 35°C (control) treatment gels scanned by UNAX Powerlook 2100XL.

**Spot#**	**Accession NO.**	**ORF**	**Gene product**	**COG Functional classification**	**MW (kDa,Theor./ Exper.)**	**pI (Theor./ Exper.)**	**Matched Peptides**	**Cov**	**Score**	**E-value**	**Visible or not visible protein spots** Vol‰ (Mean± SD)
395	ZP_06380542	ORF0089	Diaminopimelate epimerase[*Arthrospira platensis*]	Amino acid transport and metabolism Carbohydrate transport and metabolism	31.57/32.88	4.97/5.04	9	52%	94	4.00E-04	-0.66±0.020
846	ZP_03272404	ORF5508	Ribulose-bisphosphate carboxylase [*Arthrospira maxima*]	Amino acid transport and metabolism Carbohydrate transport and metabolism	53.91/53.08	6.04/5.19	32	50%	228	1.70E-17	+0.78±0.045
546	ZP_06381579	ORF3807	Glyceraldehyde-3-phosphate dehydrogenase, type I [*Arthrospira platensis*]	Amino acid transport and metabolism Carbohydrate transport and metabolism	36.89/39.08	5.41/5.90	15	44%	148	1.70E-09	+1.50±0.091
847	ZP_06381851	ORF4921	Phosphoglycerate mutase [*Arthrospira platensis*]	Amino acid transport and metabolism Carbohydrate transport and metabolism	50.68/53.08	6.01/6.68	14	31%	107	2.10E-05	+0.40±0.062
46	ZP_06382410	ORF4142	Phosphoglycerate kinase [*Arthrospira platensis*]	Amino acid transport and metabolism Carbohydrate transport and metabolism	42.36/14.81	4.92/5.03	6	25%	65	3.30E-01	- 0.64±0.052
450	ZP_06382037	ORF4701	Pfkb [*Arthrospira platensis*]	Amino acid transport and metabolism Carbohydrate transport and metabolism	36.86/34.83	4.61/4.41	8	38%	82	7.30E-03	- 0.60±0.032
747	ZP_03272404	ORF5508	Ribulose-bisphosphate carboxylase [*Arthrospira maxima*]	Amino acid transport and metabolism Carbohydrate transport and metabolism	53.91/46.33	6.04/5.78	25	42%	230	1.00E-17	- 0.64±0.040
907	ZP_06382051	ORF4691	Transketolase [*Arthrospira platensis*]	Amino acid transport and metabolism Carbohydrate transport and metabolism	72.98/62.67	5.78/6.16	14	36%	148	1.60E-09	- 0.58±0.041
928	ZP_06382051	ORF4691	Transketolase [*Arthrospira platensis*]	Amino acid transport and metabolism Carbohydrate transport and metabolism	72.98/64.76	5.78/5.75	21	45%	204	4.10E-15	- 1.10±0.087
306	YP_002248469	ORF3841	Metalloprotease ftsh [Thermodesulfovibrio yellowstonii]	Post-translational modification, protein turnover, chaperones	66.90/30.37	6.12/5.77	15	26%	89	1.30E-03	+ 0.58±0.046
60	ZP_03273444	ORF3231	Alkyl hydroperoxide reductase/ Thiol specific antioxidant/ Mal allergen [*Arthrospira maxima*]	Post-translational modification, protein turnover, chaperones	15.69/15.45	4.66/4.63	5	42%	84	3.70E-03	- 1.74±0.032
805	ZP_06380920	ORF1434	ChaperoninGroEL[*Arthrospira platensis*]	Post-translational modification, protein turnover, chaperones	57.43/50.62	5.00/4.47	24	50%	271	8.20E-22	-1.23±0.021
806	ZP_06380920	ORF1434	Chaperonin GroEL[*Arthrospira platensis*]	Post-translational modification, protein turnover, chaperones	57.43/50.62	5.00/4.67	26	53%	247	2.10E-19	- 1.18±0.035
475	ZP_06381964	ORF0151	Hypothetical protein aplap_09815 [*Arthrospira platensis*]	Signal transduction mechanisms	9.72 /6.71	7.93/6.23	9	82%	96	2.50E-04	+0.33±0.023
451	ZP_06383067	ORF0151	Hypothetical protein aplap_15418 [*Arthrospira platensis*]	Signal transduction mechanisms	21.60/35.82	5.30/6.57	14	57%	99	1.40E-04	+ 0.38±0.038
555	YP_002485086	ORF1869	Multi-sensor signal transduction histidine kinase [*Cyanothece* *sp.* PCC 7425]	Signal transduction mechanisms	85.54/39.52	5.95/4.29	11	12%	74	4.40E-02	- 0.54±0.042
480	ZP_06381260	ORF4554	Phage shock protein A, pspa [*Arthrospira platensis*]	Transcription / Signal transduction mechanisms	28.18/37.07	5.02/4.94	10	49%	90	9.30E-04	+ 0.68±0.011
283	ZP_03271593	ORF4662	Sigma 54 modulation protein/ribosomal protein S30EA [*Arthrospira maxima*]	Translation, ribosomal structure and biogenesis	23.97/29.22	7.03/6.22	9	58%	94	4.00E-04	- 0.96±0.035
78	ZP_06384870	ORF4030	Hypothetical protein aplap_24737 [*Arthrospira platensis*]	Translation, ribosomal structure and biogenesis	17.32/16.31	5.96/6.15	19	86%	254	4.10E-20	- 0.39±0.047
89	ZP_06382587	/	Hypothetical protein aplap_12998 [*Arthrospira platensis*]	Translation, ribosomal structure and biogenesis	19.30/16.69	9.47/4.11	14	72%	187	2.10E-13	- 0.48±0.056
314	ZP_06384062	ORF0361	Peptidase S8 and S53 subtilisin kexin sedolisin [*Arthrospira platensis*]	Translation, ribosomal structure and biogenesis	44.24/30.34	4.59/4.53	16	45%	153	5.20E-10	- 0.64±0.042
423	ABV01983	ORF4633	Cpch [*Arthrospira platensis*]	Translation, ribosomal structure and biogenesis	30.85/33.93	7.82/6.29	25	60%	295	3.30E-24	- 0.42±0.033
424	ZP_03272395	ORF5516	Conserved hypothetical protein [*Arthrospira maxima*]	Translation, ribosomal structure and biogenesis	41.12/34.02	6.04/6.08	13	50%	205	3.30E-15	- 0.58±0.034
390	ZP_06382427	ORF2155	Phycobilisome linker polypeptide [*Arthrospira platensis*]	Energy metabolism (photosynthesis, respiratory electron transport)	29.45/33.10	9.25/6.61	31	76%	313	5.20E-26	+ 0.69±0.056
137	ZP_03271326	ORF5159	Phycobilisome protein [*Arthrospira maxima*]	Energy metabolism (photosynthesis, respiratory electron transport)	17.44/19.04	4.89/4.93	16	62%	148	1.70E-09	+ 0.50±0.066
140	ZP_03271568	ORF4634	Phycocyanin, alpha subunit [*Arthrospira maxima*]	Energy metabolism (photosynthesis, respiratory electron transport)	17.70/19.59	5.82/6.11	11	75%	91	8.10E-04	+ 0.64±0.043
147	ZP_03271568	ORF4634	Phycocyanin, alpha subunit [*Arthrospira maxima*]	Energy metabolism (photosynthesis, respiratory electron transport)	17.70/20.47	5.82/5.83	12	75%	81	7.60E-03	+ 0.57±0.054
47	ZP_03271327	ORF4103	Allophycocyanin, beta subunit [*Arthrospira maxima*]	Energy metabolism (photosynthesis, respiratory electron transport)	17.43/14.81	6.26/5.74	14	84%	165	3.30E-11	- 0.43±0.007
318	ZP_06382427	ORF2155	Phycobilisome linker polypeptide [*Arthrospira platensis*]	Energy metabolism (photosynthesis, respiratory electron transport)	29.45/30.46	9.25/4.26	15	53%	160	1.00E-10	- 0.52±0.062
410	ZP_06382427	ORF2155	Phycobilisome linker polypeptide [*Arthrospira platensis*]	Energy metabolism (photosynthesis, respiratory electron transport)	29.45/33.46	9.25/6.41	15	54%	186	2.60E-13	- 0.38±0.032
168	ZP_06382815	ORF4792	DNA starvation/stationary phase protection protein Dps [*Arthrospira platensis*]	Inorganic ion transport and metabolism	19.67/23.51	4.93/6.24	10	70%	81	8.50E-03	+ 0.74±0.052
423	YP_001999788	ORF2422	Preprotein translocase subunit seca [*Mycoplasma arthritidis*]	Intracellular trafficking and secretion	99.38/34.57	5.10/5.15	21	28%	76	2.60E-02	+ 0.59±0.034
540	ZP_06381727	ORF2131	Beta-lactamase-like protein [*Arthrospira platensis*]	General function prediction only	34.13/39.06	5.12/5.47	14	40%	148	1.70E-09	+ 0.80±0.033
10	ZP_06383388	ORF2597	Short-chain dehydrogenase/reductase SDR [*Arthrospira platensis*]	General function prediction only	19.51/27.67	5.54/6.38	12	64%	129	1.30E-07	- 0.40±0.028
251	ZP_06381424	ORF1751	NAD-dependent epimerase/dehydratase [*Arthrospira platensis*]	Cell envelope biogenesis, outer membrane	23.63/28.08	5.15/5.75	12	70%	184	4.10E-13	- 1.70±0.037
188	ZP_06380822	ORF1456	Pentapeptide repeat-containing protein [*Arthrospira platensis*]	Function unknown	19.86/25.09	5.13/5.67	16	58%	117	2.10E-06	+ 0.67±0.091
181	YP_912068	/	Hypothetical protein Cpha266_1623 [*Chlorobium phaeobacteroides*]	Function unknown	31.01/24.60	6.56/4.97	12	43%	84	3.90E-03	+ 0.55±0.12
391	ZP_03271502	ORF4558	Conserved hypothetical protein [*Arthrospira maxima*]	Function unknown	27.65/33.16	4.75/4.53	11	49%	88	1.80E-03	+ 0.32±0.029
275	ZP_06380972	ORF1961	Ppic-type peptidyl-prolyl cis-trans isomerase	Function unknown	31.57/28.94	5.27/4.94	22	74%	190	1.00E-13	+ 0.52±0.032

Note: (1) In “Visible or not visible protein spots” column, “+”indicates the visible protein spots in the 15°C treatment gels, while not visible in the 35°C control gels. “-” indicates the visible protein spots in the 35°C control gels, while not visible in the 15°C treatment gels.

(2) The relative abundance (Vol‰,Mean± SD) of “visible” protein calculated by the ratio of volume of each protein spot to volume of total protein spots in the whole gel.

**Table 2:** The visible or not visible protein spots at 45°C vs 35°C (control) treatment gels scanned by UNAX Powerlook 2100XL.

**Spot#**	**Accession NO.**	**ORF**	**Gene product**	**COG Functional classification**	**MW (kDa,Theor./ Exper.)**	**pI (Theor./Exper.)**	**Matched Peptides**	**Cov**	**Score**	**E-value**	**Visible or not visible protein spots** Vol‰ (Mean± SD)
648	ZP_03273305	ORF0278	Pyridoxal-5'-phosphate-dependent protein beta subunit [*Arthrospira maxima*]	Amino acid transport and metabolism	34.84/37.95	5.28/5.23	26	69%	241	8.30E-19	+0.99±0.031
331	ZP_06381552	ORF0785	Ribose-5-phosphate isomerase A [*Arthrospira platensis*]	Carbohydrate transport and metabolism	25.13/28.28	4.96/5.04	10	46%	105	3.30E-05	+ 0.50±0.045
1120	ZP_06381999	ORF0376	Transketolase domain protein [*Arthrospira platensis*]	Carbohydrate transport and metabolism	69.60/64.11	5.19/5.52	41	64%	297	2.10E-24	+ 0.36±0.085
129	ZP_03273475	ORF1368	Redoxin domain protein [*Arthrospira maxima*]	Post-translational modification, protein turnover, chaperones	19.78/20.11	4.88/5.03	10	49%	119	1.30E-06	-0.75±0.037
224	ZP_03275641	ORF0073	Ribosomal protein L6 [*Arthrospira maxima*]	Translation, ribosomal structure and biogenesis	19.69/25.13	10.25/5.63	11	40%	104	4.20E-05	+0.60±0.025
433	ZP_06380559	ORF1074	Elongation factor TS [*Arthrospira platensis*]	Translation, ribosomal structure and biogenesis	24.63/31.08	5.74/5.90	21	77%	155	3.30E-10	+0.39±0.019
283	ZP_03271593	ORF4662	Sigma54 modulation protein/ribosomal protein S30EA[*Arthrospira maxima*]	Translation, ribosomal structure and biogenesis	23.97/29.22	7.03/6.22	9	58%	94	4.00E-04	-0.96±0.068
377	ZP_06380559	ORF1074	Elongation factor TS [*Arthrospira platensis*]	Translation, ribosomal structure and biogenesis	24.63/32.19	5.74/6.13	7	53%	75	3.40E-02	-0.83±0.076
118	ZP_03271568	ORF4634	Phycocyanin, alpha subunit [*Arthrospira maxima*]	Energy production and conversion	17.70/18.27	5.82/6.00	11	74%	120	1.00E-06	+1.67±0.031
143	ZP_03271568	ORF4634	Phycocyanin, alpha subunit [*Arthrospira maxima*]	Energy production and conversion	17.70/19.60	5.82/6.05	14	86%	114	4.20E-06	+0.35±0.043
70		/	Phycocyanin, alpha subunit [*Arthrospira maxima*]	Energy production and conversion	17.70/16.05	5.82/4.60	10	74%	98	1.70E-05	+0.44±0.055
517	ABB84420	ORF4632	Cpci [*Arthrospira platensis*]	Energy production and conversion	32.79/33.33	8.33/5.68	17	51%	127	2.10E-07	+0.46±0.026
47	ZP_03271327	ORF4103	Allophycocyanin, beta subunit [*Arthrospira maxima*]	Energy production and conversion	17.43/14.81	6.26/5.74	14	84%	165	3.30E-11	-0.43±0.080
185	ZP_06383806	ORF0728	Hypothetical protein aplap_19246 [*Arthrospira platensis*]	General function prediction only	22.95/23.52	4.99/5.22	23	70%	166	2.60E-11	+0.46±0.045
886	ZP_06384147	ORF2097	Von Willebrand factor, type A [*Arthrospira platensis*]	General function prediction only	37.19/43.74	5.81/5.77	19	57%	167	2.10E-11	+0.86±0.023
934	ZP_06381540	ORF1251	Hypothetical protein aplap_07632 [*Arthrospira platensis*]	General function prediction only	67.41/65.34	5.66/6.21	19	42%	166	2.60E-11	-1.63±0.019
158	ZP_06383329	ORF4728	Hypothetical protein aplap_16770 [*Arthrospira platensis*]	Function unknown	19.34/21.31	5.30/5.83	9	59%	120	1.00E-06	+0.35±0.036
78	ZP_06384870	ORF4030	Hypothetical protein aplap_24737 [*Arthrospira platensis*]	Function unknown	17.32/16.31	5.96/6.14	19	86%	254	4.10E-20	-0.39±0.098
98	ZP_06384870	ORF4030	Hypothetical protein aplap_24737 [*Arthrospira platensis*]	Function unknown	17.32/17.19	5.96/6.50	18	82%	214	4.10E-16	-0.53±0.022
339	ZP_06384062	ORF0361	Peptidase S8 and S53 subtilisin kexin sedolisin [*Arthrospira platensis*]	Function unknown	44.24/31.22	4.59/4.65	16	44%	200	1.00E-14	-0.77±0.054

Note: (1) In “Visible or not visible protein spots” column, “+”indicates the visible protein spots in the 45°C treatment gels, while not visible in the 35°C control gels. “-” indicates the visible protein spots in the 35°C control gels, while not visible in the 45°C treatment gels.

(2) The relative abundance (Vol‰, Mean± SD) of “visible” protein calculated by the ratio of volume of each protein spot to volume of total protein spots in the whole gel.

**Figure 3:**
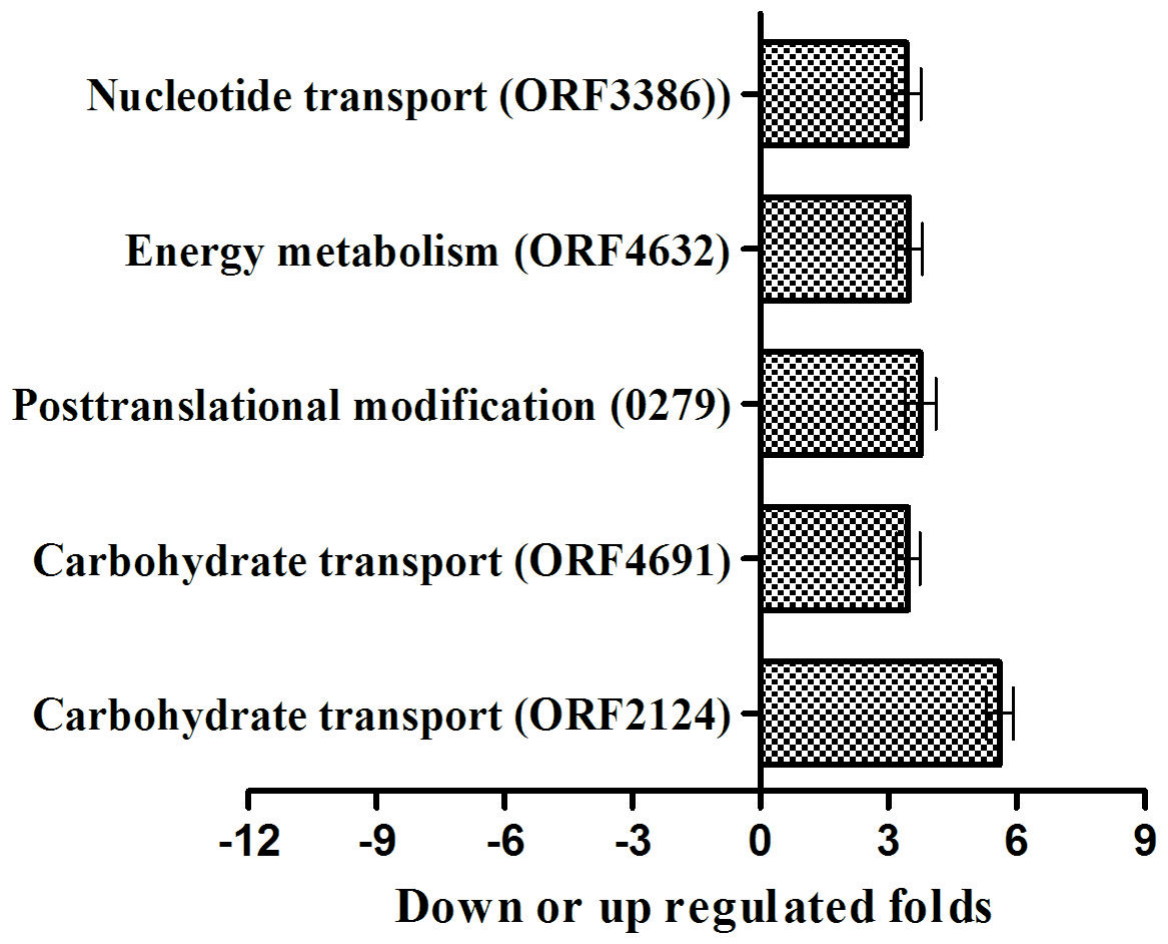
The differential protein spots between high temperature (45°C) and low temperature (15°C) treatment. Note: The differential protein spots with >3.0-fold changes in low temperature (15°C) treatment compared to high temperature (45°C) treatment.

By comparing the differentially expressed protein in the high and low temperature treatments, 5 protein spots were found to express differentially by more than 3-fold higher at 15°C than at 45°C (Figure 3). In addition, 8 protein spots were solely visible at 15°C, while 6 protein spots were solely visible at 45°C (Table 3). In total, 122 differentially expressed protein spots were obtained from the control (35°C), low (15°C) and high (45°C) temperature groups (see Table 3 A, 3B and 3C).

**Table 3:** The visible or not visible protein spots at 15°C vs 45°C treatment gels scanned by UNAX Powerlook 2100XL.

**Spot#**	**Accession NO.**	**ORF**	**Gene product**	**COG Functional classification**	**MW (kDa,Theor./ Exper.)**	**pI (Theor./Exper.)**	**Matched Peptides**	**Cov**	**Score**	**E-value**	**Visible or not visible protein spots Vol‰ (Mean± SD)**
516	ZP_06381532	ORF1242	Cysteine synthase[*Arthrospira platensis*]	Amino acid transport and metabolism	34.53/33.33	5.92/6.38	19	44%	150	1.00E-09	-0.58±0.022
987	ZP_06382051	ORF4691	Transketolase [*Arthrospira platensis*]	Carbohydrate transport and metabolism	72.98/77.27	5.78/5.53	16	29%	104	4.20E-05	+0.82±0.038
669	ZP_03272326	ORF5578	UBA/tdIF-type NAD/FAD binding protein [*Arthrospira maxima*]	Coenzyme metabolism	43.01/42.01	5.06/4.99	15	50%	125	3.30E-07	+0.78±0.047
256	ZP_05256482	ORF1829	Radical SAM domain-containing protein [Bacteroides]	Coenzyme metabolism	36.20/28.14	6.21/6.59	10	35%	77	2.10E-02	+0.49±0.035
859	ZP_03275668	ORF4248	Cysteinyl-trna synthetase [*Arthrospira maxima*]	Translation, ribosomal structure and biogenesis	55.01/53.51	5.87/6.50	19	38%	76	2.80E-02	+0.57±0.028
514	ZP_06380559	ORF1074	Elongation factor TS [*Arthrospira platensis*]	Translation, ribosomal structure and biogenesis	24.63/33.40	5.74/5.90	18	70%	148	1.70E-09	-0.33±0.027
332	ZP_03273564	ORF2382	Ferredoxin [*Arthrospira maxima*]	Energy metabolism (photosynthesis, respiratory electron transport)	27.02/31.29	5.68/5.78	14	47%	101	8.30E-05	+0.59±0.034
332*	ZP_06380716	ORF2382	Bidirectional hydrogenase complex protein hoxu [*Arthrospira platensis*]	Energy metabolism (photosynthesis, respiratory electron transport)	27.00/31.29	5.54/5.78	17	68%	143	5.20E-09	+0.59±0.046
478	ZP_06382427	ORF2155	Phycobilisome linker polypeptide [*Arthrospira platensis*]	Energy metabolism (photosynthesis, respiratory electron transport)	29.45/32.15	9.25/5.85	25	70%	206	2.60E-15	-1.03±0.057
772	ZP_06381274	ORF0324	UDP-glucose/GDP-mannose dehydrogenase [*Arthrospira platensis*]	Cell envelope biogenesis, outer membrane	49.93/47.00	5.18/5.14	19	47%	110	1.00E-05	+0.50±0.050
941	ZP_03275087	ORF0324	Nucleotide sugar dehydrogenase [*Arthrospira maxima*]	Cell envelope biogenesis, outer membrane	34.72/47.54	5.26/5.16	30	72%	249	1.30E-19	-1.02±0.086
118	ZP_06382587		Hypothetical protein aplap_12998 [*Arthrospira platensis*]	Function unknown	19.30/17.10	9.47/6.55	15	74%	183	5.20E-13	+0.66±0.033
183	ZP_03274017	ORF1894	Uncharacterized protein/domain associated with gtpase-like protein [*Arthrospira maxima*]	Function unknown	20.09/23.37	5.74/5.93	5	32%	78	1.80E-02	-0.50±0.049
425	ZP_06384062	ORF0361	Peptidase S8 and S53 subtilisin kexin sedolisin [*Arthrospira platensis*]	Function unknown	44.24/30.81	4.59/4.46	24	54%	156	2.60E-10	-0.56±0.021

Note: (1) In “Visible or not visible protein spots” column, “+”indicates the solely visible protein spots in the 15°C treatment gels, and “-”indicates the solely visible protein in the 45°C treatment gels.

(2) The relative abundance (Vol‰, Mean± SD) of “visible” protein calculated by the ratio of volume of each protein spot to volume of total protein spots in the whole gel.

### PMF of the differentially expressed protein spots and MALDI-TOF MS analysis

The TwoClassDif (RVM-T test) method with a cutoff of *p*-value <0.05 and FDR <0.05 was applied to analyze the three different temperature treatments. Only those with reproducibility and the fold change with >3.0-fold threshold were considered to be differentially expressed protein spots. The confidence in the peptide mass fingerprinting matches (*p*<0.05) was based on the MOWSE Score more than 75. The mass spectrum of identified subset protein spots is shown in Figure S2. Using MASCOT searching, 122 positively expressed protein spots were categorized into several groups according to their functions, of which 116 proteins were demonstrated to have high homology with the *Arthrospira platensis* genus. However, the other 6 proteins (YP_002248469, YP_002485086, ZP_04040440, YP_001999788, YP_912068, ZP_05256482) had high homology with other prokaryotes, which belonged to *Hermodesulfovibrio yellowstonii, Cyanothece* *sp.* PCC 7425, *Meiothermus ruber DSM 1279, Mycoplasma arthritidis, Chlorobium phaeobacteroides and Bacteroides*, respectively. All of the above prokaryotes are high temperature-tolerant bacteria with the exception of *Cyanothece* *sp.* PCC 7425 (see Tables S3A, S3B and S3C).

**Figure 4:**
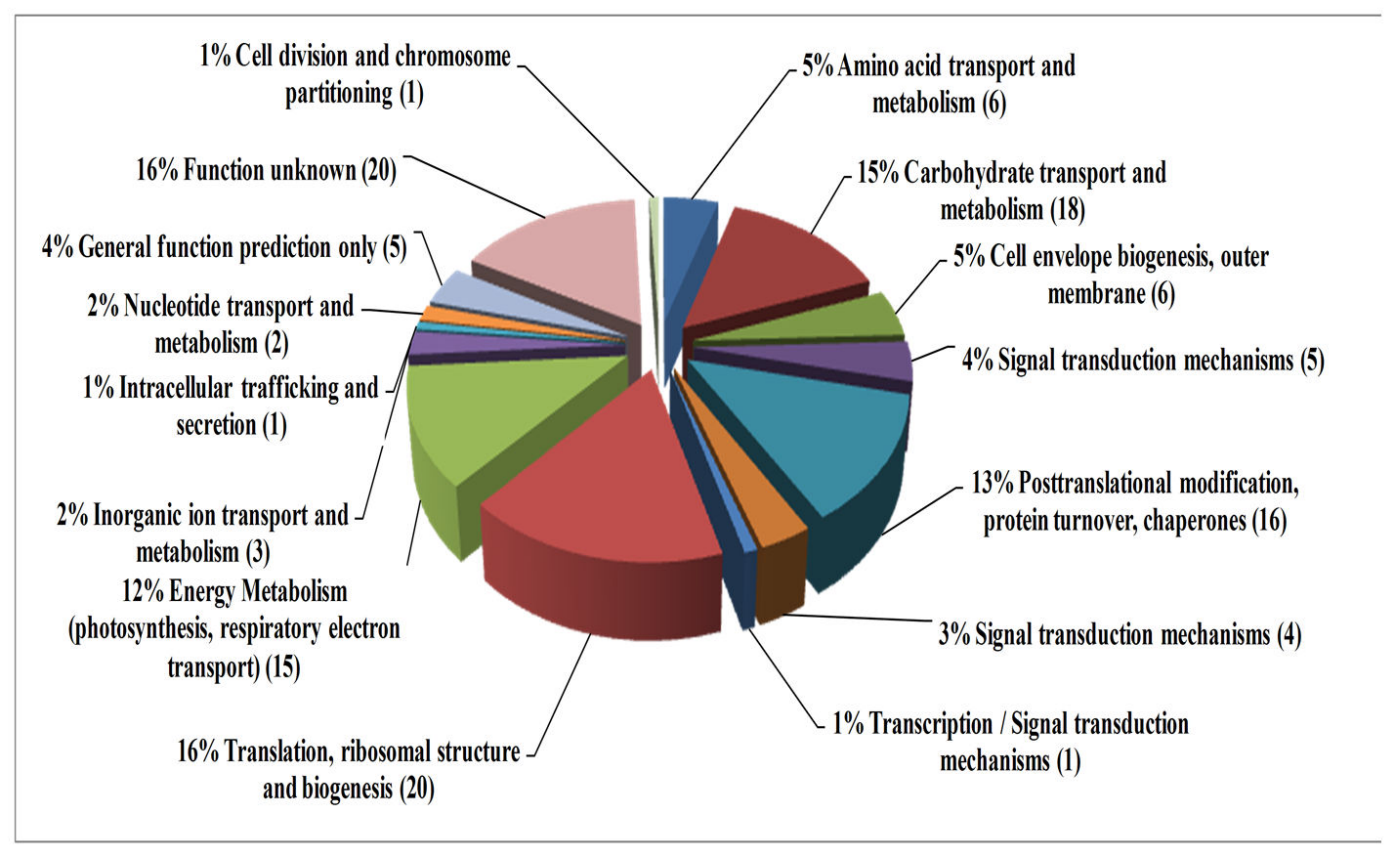
COG functional classification of 122 positively differential expression proteins.

By comparing the theoretical and actual values of Mw and pI of Matched Proteins acquired by MS and Gel Map Analytical Software, we performed COG function prediction and classified the 122 positively expressed proteins into 15 functional categories. All kinds of functional protein spots differentially expressed at different temperatures as summarized in Table 4, and the proportion of the different kinds of functional protein is shown in Figure 4 on the basis of proven high credibility. At 15°C, the highest proportion of gene functions was related to energy metabolism (photosynthesis, respiratory and electron transport), carbohydrate transport and metabolism, post-translational modification, protein turnover, chaperones and coenzyme metabolism; in addition there were some unknown function genes. Similarly at 45°C, the highest proportion of gene functions was concerned with energy metabolism (photosynthesis, respiratory and electron transport), while some protein functions were related to carbohydrate transport, metabolism and translation, ribosomal structure and biogenesis. In summary, a large portion of the differentially expressed protein spots that responded to high or low temperatures were related to phycobilisome (PBS), which could affect algal photosynthesis and energy metabolism.

**Table 4:** Functional classification of the differential protein spots in different temperature treatments.

NO.	COG classification	Total number at 15°C	Total number at 35°C	Total number at 45°C	Total number
1	Amino acid transport and metabolism	3	1	2	6
2	Carbohydrate transport and metabolism	7	7	4	18
3	Cell envelope biogenesis, outer membrane	4	1	1	6
4	Coenzyme metabolism	5	/	/	5
5	Post-translational modification, protein turnover, chaperones	7	7	/	14
6	Signal transduction mechanisms	3	1	/	4
7	Transcription / Signal transduction mechanisms	1	/	/	1
8	Translation, ribosomal structure and biogenesis	3	10	3	16
9	Energy Metabolism (photosynthesis, respiratory electron transport)	8	6	7	21
10	Inorganic ion transport and metabolism	2	/	1	3
11	Intracellular trafficking and secretion	1	/	/	1
12	Nucleotide transport and metabolism	1	1	/	2
13	General function prediction only	1	1	2	4
14	Function unknown	9	5	6	20
15	Cell division and chromosome partitioning	/	1	/	1
16	Total function proteins	54	42	26	122

### qRT-PCR analyses of the differentially expressed genes

#### The purity and concentration of total RNA

Based on agarose gel electrophoresis determination, the extracted RNA in the low (15°C), control (35°C) and high (45°C) temperature treatments showed good integrity and high purity, and had no degradation (see Figure S3). The OD_260_/OD_280_ triplicate values were 2.09, 2.03 and 2.04, and the OD_260_/_230_ triplicate values were 2.08, 2.24 and 2.19 for the low (15°C), control (35°C) and high (45°C) temperature groups, respectively. The RNA concentrations were 241.2, 213.6 and 209.5 ng/µL in the low (15°C), control (35°C) and high (45°C) temperature treatments, respectively. Both OD_260_/OD_280_ and OD_260_/_230_ values were greater than 2.0, demonstrating no contamination of RNA by proteins, salts or carbohydrates.

#### Standard curve for qRT-PCR

We selected a total of 38 genes for further validation, of which 23 genes were found to be significantly different between the 15°C treatment and control (35°C), 13 genes between the 45°C treatment and control, and 2 genes between the 15 and 45°C treatments (ORF4248 up-regulated at 15°C and ORF0324 down-regulated at 45°C). The information for annotation and metabolic pathway of the 38 genes is summarized in Table 5, and the consistency between their transcription level and gene expression was confirmed by qRT-PCR. All of the melting curves showed a single peak in the tested range of standard curves, suggesting good specificity (standard curves not shown in Figure). A non-detectable fluorescence signal in negative and blank controls indicated that the reaction system was not contaminated during the experimental procedures. The calibration curves for 16S rRNA as endogenous control and target genes, regression coefficients and amplification efficiencies were between 98.2-99.8% and 81.1-117.2%, which indicated efficient amplification with high specificity (see Table S2).

**Table 5:** Annotation and proposed metabolic pathways of the 38 differential protein spots obtained from the MS-searching.

Spot	ORF	Accession NO.	Gene Product	COG Functional classification	MW (kDa,Theor./ Exper.)	pI (Theor./Exper.)	Matched Peptides	Cov	MASCT Score	E-value	Fold change Mean± SD
859	ORF4248	ZP_03275668	Cysteinyl-trna synthetase [Arthrospira maxima]	Translation, ribosomal structure and biogenesis	55.01/53.51	5.87/6.50	19	38%	76	2.80E-02	6.36±0.41
433	ORF1074	ZP_06380559	Elongation factor TS [Arthrospira platensis]	Translation, ribosomal structure and biogenesis	24.63/31.08	5.74/5.90	21	77%	155	3.30E-10	/
224	ORF0073	ZP_03275641	Ribosomal protein L6 [Arthrospira maxima]	Translation, ribosomal structure and biogenesis	19.69/25.13	10.25/5.63	11	40%	104	4.20E-05	/
283	ORF4662	ZP_03271593	Sigma 54 modulation protein/ribosomal protein S30EA [Arthrospira maxima]	Translation, ribosomal structure and biogenesis	23.97/29.22	7.03/6.22	9	58%	94	4.00E-04	/
517	ORF4632	ABB84420	Cpci [Arthrospira platensis]	Energy production and conversion	32.79/33.33	8.33/5.68	17	51%	127	2.10E-07	/
143	ORF4634	ZP_03271568	Phycocyanin, alpha subunit [Arthrospira maxima]	Energy production and conversion	17.70/19.60	5.82/6.05	14	86%	114	4.20E-06	/
564	ORF4633	ZP_06380687	Phycobilisome linker polypeptide [Arthrospira platensis]	Energy production and conversion	30.87/34.48	7.82/6.60	30	71%	338	1.70E-28	3.09±0.28
486	ORF2155	ZP_06382427	Phycobilisome linker polypeptide [Arthrospira platensis]	Energy production and conversion	29.45/32.42	9.25/6.25	25	70%	237	2.10E-18	5.39±0.35
941	ORF0324	ZP_03275087	Nucleotide sugar dehydrogenase [Arthrospira maxima]	Cell envelope biogenesis, outer membrane	34.72/47.54	5.26/5.16	30	72%	249	1.30E-19	/
868	ORF5692	ZP_06383693	Phosphateuridyltransferase/glucosamine phosphateacetyl transferase[Arthrospira platensis]	Cell envelope biogenesis, outer membrane	50.14/54.88	5.74/6.18	23	50%	235	3.30E-18	3.38±0.27
213	ORF1751	ZP_06381424	NAD-dependent epimerase/dehydratase [Arthrospira platensis]	Cell envelope biogenesis, outer membrane	23.63/26.06	5.15/5.49	23	78%	231	8.30E-18	3.19±0.28
160	ORF4935	ZP_06381012	Acetolactate synthase regulatory	Amino acid transport and metabolism	19.26/22.13	6.62/6.80	14	64%	154	4.20E-10	3.10±0.22
535	ORF3909	ZP_06382239	Aspartate-semialdehyde dehydrogenase [Arthrospira platensis]	Amino acid transport and metabolism	37.27/38.57	5.38/5.72	22	64%	209	1.30E-15	3.43±0.29
492	ORF1242	ZP_06381042	Cysteine synthase A [Arthrospira platensis]	Amino acid transport and metabolism	34.84/37.37	5.33/5.85	23	67%	151	8.30E-10	3.39±0.15
648	ORF0278	ZP_03273305	Pyridoxal-5'-phosphate-dependent protein beta subunit [Arthrospira maxima]	Amino acid transport and metabolism	34.84/37.95	5.28/5.23	26	69%	241	8.30E-19	/
664	ORF5578	ZP_03272326	UBA/THIF-type NAD/FAD binding protein [Arthrospira maxima]	Coenzyme metabolism	43.01/41.67	5.06/5.20	15	49%	147	2.10E-09	3.75±0.28
235	ORF4133	ZP_03275242	Pyridoxal phosphate biosynthetic protein pdxj [Arthrospira maxima]	Coenzyme metabolism	26.10/27.02	5.30/5.52	7	47%	114	4.20E-06	4.69±0.43
536	ORF4139	ZP_06381750	Tetrahydrofolatede Hydrogenase/ cyclohydrolase [Arthrospira platensis]	Coenzyme metabolism	30.92/38.66	6.01/6.79	10	32%	76	2.50E-02	5.49±0.42
747	ORF5508	ZP_03272404	Ribulose-bisphosphate carboxylase[Arthrospira maxima]	Carbohydrate transport and metabolism	53.91/46.33	6.04/5.78	25	42%	230	1.00E-17	/
1120	ORF0376	ZP_06381999	Transketolase domain protein [Arthrospira platensis]	Carbohydrate transport and metabolism	69.60/64.11	5.19/5.52	41	64%	297	2.10E-24	/
928	ORF4691	ZP_06382051	Transketolase [Arthrospira platensis]	Carbohydrate transport and metabolism	72.98/64.76	5.78/5.75	21	45%	204	4.10E-15	/
648	ORF3656	ZP_06380758	Fructose-1,6-bisphosphate Aldolase [Arthrospira platensis]	Carbohydrate transport and metabolism	38.99/41.19	5.60/4.49	13	33%	168	1.60E-11	/
507*	ORF4543	ZP_06383180	Hypothetical protein aplap15993 [Arthrospira platensis]	Cell division and chromosome partitioning	38.08/38.00	4.59/4.65	21	58%	190	1.00E-13	-5.07±0.43
156	ORF1368	ZP_03273475	Redoxin domain protein[Arthrospira maxima]	Post-translational modification, protein turnover, chaperones	19.78/21.51	4.88/5.18	15	66%	133	5.20E-08	5.85±0.43
916	ORF1976	ZP_03275215	Chaperonin GroEL [Arthrospira maxima]	Post-translational modification, protein turnover, chaperones	58.21/57.65	5.00/5.25	28	56%	183	5.20E-13	3.84±0.29
275	ORF2281	ZP_06380867	Peptidyl-prolyl cis-trans isomerase, cyclophilin type [Arthrospira platensis]	Post-translational modification, protein turnover, chaperones	23.92/28.93	4.74/4.49	6	36%	75	3.60E-02	/
805	ORF1434	ZP_06380920	ChaperoninGroEL[Arthrospira platensis]	Post-translational modification, protein turnover, chaperones	57.43/50.62	5.00/4.47	24	50%	271	8.20E-22	/
210	ORF2861	ZP_06382415	Stress protein [Arthrospira platensis]	Signal transduction mechanisms	22.20/26.12	4.93/5.23	14	78%	111	8.30E-06	3.82±0.19
170	ORF4792	ZP_06382815	DNA starvation/stationary phase protection protein Dps [Arthrospira platensis]	Inorganic ion transport and metabolism	19.67/23.83	4.93/5.04	13	70%	110	8.30E-06	3.07±0.27
165	ORF1911	ZP_06383116	Adenylylsulfate kinase [Arthrospira platensis]	Inorganic ion transport and metabolism	19.90/21.51	5.22/5.18	12	66%	149	1.30E-09	3.32±0.22
934	ORF1251	ZP_06381540	Hypothetical protein aplap_07632 [Arthrospira platensis]	General function prediction only	67.41/65.34	5.66/6.21	19	42%	166	2.60E-11	/
137	ORF5159	ZP_03271326	Phycobilisome protein [Arthrospira maxima]	Energy metabolism (photosynthesis, respiratory electron transport	17.44/19.04	4.89/4.93	16	62%	148	1.70E-09	/
961	ORF2208	ZP_06383545	Hypothetical protein aplap_17874 [Arthrospira platensis]	Function unknown	18.76/65.83	5.42/5.58	13	77%	94	4.30E-04	4.10±0.28
413	ORF0361	ZP_06384062	Peptidase S8 and S53 subtilisin kexin sedolisin [Arthrospira platensis]	Function unknown	44.24/34.26	4.59/4.57	22	49%	166	2.60E-11	5.25±0.48
275	ORF1961	ZP_06380972	Ppic-type peptidyl-prolyl cis-trans isomerase	Function unknown	31.57/28.94	5.27/4.94	22	74%	190	1.00E-13	3.12±0.24
216	ORF1456	ZP_06380822	Pentapeptide repeat-containing protein [Arthrospira platensis]	Function unknown	19.86/24.68	5.13/5.44	16	58%	125	3.30E-07	3.32±0.29
680	ORF4030	ZP_06384870	Hypothetical protein aplap_24737 [Arthrospira platensis]	Function unknown	17.32/15.87	5.96/6.23	20	93%	224	4.20E-17	3.19±0.21
188	ORF2593	ZP_03274149	Beta-propeller repeat TECPR[Arthrospira maxima]	Function unknown	22.58/25.41	4.81/4.72	9	34%	89	1.30E-03	/

Note: (1) Fold change indicates Mean± SD; (2) “/ ” indicates the visible or not visible protein spots scanned by UNAX Powerlook 2100X

#### The relative transcription level of genes using qRT-PCR

**Figure 5:**
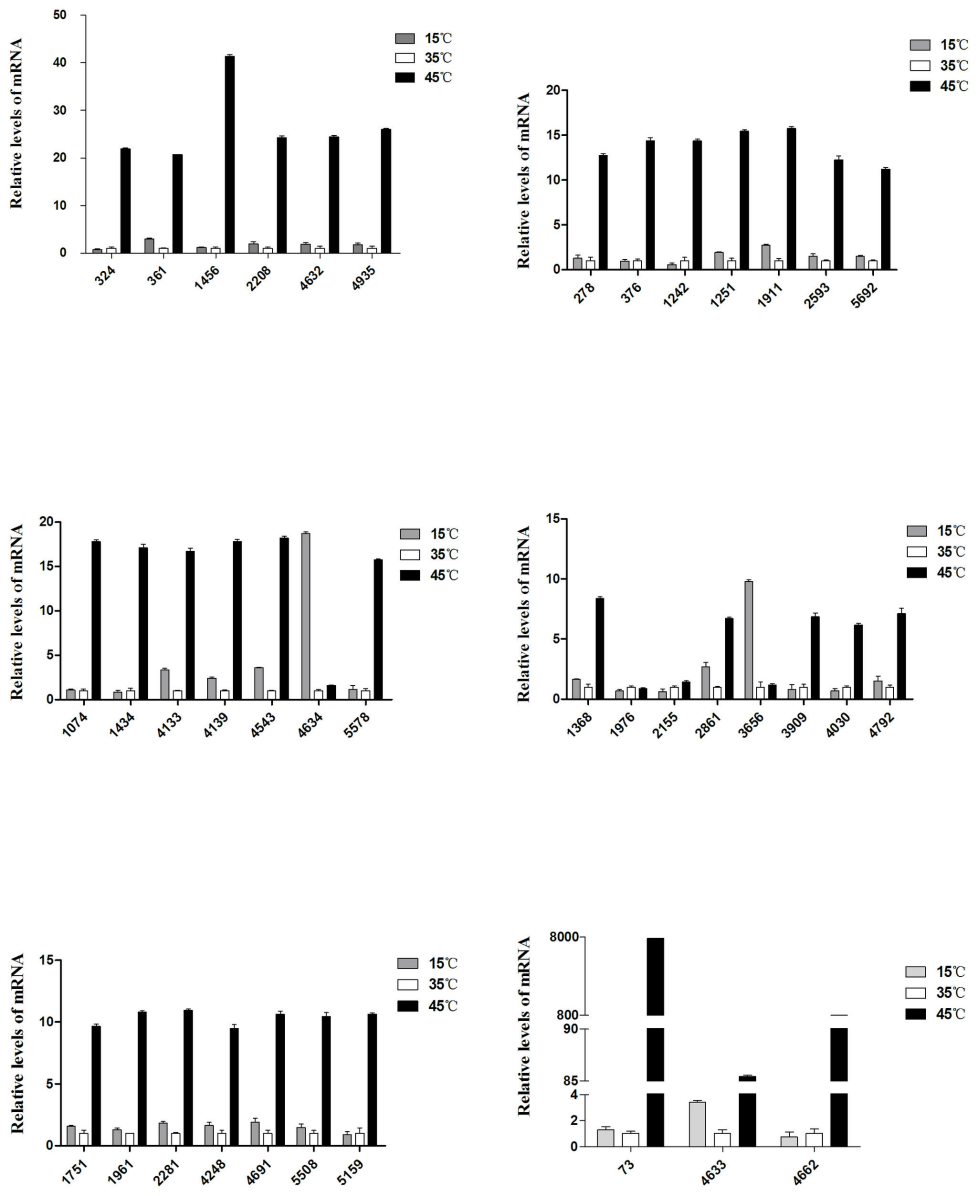
The differential expression of the selected 38 genes for the control and the temperature-stress treatment group by qRT-PCR.

The mRNA relative transcription levels of positively expressed proteins detected by qRT-PCR for the control, high and low temperature treatments are shown in Figure 5. At 15°C, there were 23 genes with >3-fold differential expression, among which 16 genes were up-regulated and 7 genes were down-regulated on the protein level. However, the transcription levels of 17 genes were up-regulated, while those of 6 genes were down-regulated. Of the 17 up-regulated genes, the genes with >2-fold transcription level were ORF3656, ORF4133, ORF2861, ORF4139 and ORF361. The 6 down-regulated genes all exceeded a one-fold change. At 45°C, the transcription levels of 10 genes were up-regulated more than 3-fold, while those of 3 genes (ORF73, ORF4543 and ORF1251) were down-regulated in 13 differentially expressed proteins. At the transcription level, 10 genes were up-regulated more than 3-fold, while ORF4634 and ORF2155 displayed less than a 2-fold change. When comparing low with high temperature treatments, the transcription and expression levels of ORF324 were consistent, but those of ORF4248 were inconsistent, i.e., the transcription of ORF4248 was down-regulated and the translation was up-regulated. More interestingly, the fold changes of ORF4633 and ORF73 with up-regulated transcription levels were more than 85-fold and 7867-fold, respectively. Of the 38 target genes, the protein expression of 26 genes was consistent with gene transcription, representing 68.4% of the total target genes. The remaining 12 genes showed inconsistent protein expression with transcription level, representing 31.6% of the total target genes. A comparison of transcription and protein expression of the target genes is summarized in Table 6.

**Table 6:** The comparison between qPCR and proteomic analysis result.

**Accession NO.**	**ORF**	**qPCR results 15-2** ^**-**⊿⊿**Ct**^ ** /35-2** ^**-**⊿⊿**Ct**^	**Fold change from proteomic results**	**Consistency**
ZP_06381424	1751	22.9/14.6 ↑	↑ 3.19	Y
ZP_06380972	1961	14.0/10.6 ↑	↑ 3.12	Y
ZP_06382815	4792	50.6/33.2 ↑	↑ 3.07	Y
ZP_06383693	5692	30.4/20.3 ↑	↑ 3.38	Y
ZP_06383545	2208	1.97/1.00 ↑	↑ 4.1	Y
ZP_03275242	4133	0.37/0.11 ↑	↑ 4.69	Y
ZP_06381012	4935	0.60/0.34 ↑	↑ 3.1	Y
ZP_03272326	5578	5.30e-05/4.59e-05 ↑	↑ 3.75	Y
ZP_06380920	1434	0.87/1.00 ↓	↓ -	Y
ZP_06382415	2861	11.1/4.09 ↑	↑ 3.82	Y
ZP_06381750	4139	2.24/0.94 ↑	↑ 5.49	Y
ZP_03271593	4662	2.38e-05/3.26e-05 ↓	↓ -	Y
ZP_03273475	1368	5.78/3.43 ↑	↑ 3.45	Y
ZP_06384062	0361	1.46/0.50 ↑	↑ 5.25	Y
ZP_06381042	1242	0.56/1.00 ↓	↑ 3.39	N
ZP_06380867	2281	25.1/13.6 ↑	↓ -3.12	N
ZP_06382239	3909	4.41/5.42 ↓	↑ 3.4	N
ZP_03275215	1976	0.56/1.35 ↓	↑ 3.84	N
ZP_06380758	3656	3.02/0.31 ↑	↓ -4.39	N
ZP_03271326	5159	2.51/2.77 ↓	↑ +	N
ZP_03274149	2593	2.88/1.90 ↑	↓ +	N
ZP_06382051	4691	2.55/1.33 ↑	↓ +	N
ZP_03272404	5508	25.5/17.3 ↑	↓ +	N
	ORF	**qPCR results 45-2^-^** ^⊿⊿^ **^C^^t^**/**35-2** ^**-**⊿⊿**Ct**^	**Fold change from proteomic results**	**Consistency**
ZP_03275641	0073	0.80/0.0001 ↑	↑ +	Y
ZP_06383116	1911	25.3/1.61 ↑	↑ 3.32	Y
ABB84420	4632	0.50/0.02 ↑	↑ +	Y
ZP_03271568	4634	0.41/0.26 ↑	↑ +	Y
ZP_06380822	1456	14.0/0.34 ↑	↑ 3.32	Y
ZP_06382427	2155	0.17/0.12 ↑	↑ 5.39	Y
ZP_03273305	0278	108/8.49 ↑	↑ +	Y
ZP_06381999	0376	14.7/1.02 ↑	↑ +	Y
ZP_0638487	4030	193/31.2 ↑	↑ 3.19	Y
ZP_06380687	4633	5.58/0.07 ↑	↑ 3.09	Y
ZP_06380559	1074	17.8/1.00 ↑	↑ +	Y
ZP_06383180	4543	19.6/1.08 ↑	↓ -5.07	N
ZP_06381540	1251	15.5/1.00 ↑	↑ -	N
	ORF	qPCR results 45-2^-⊿⊿Ct^/15-2^-⊿⊿Ct^	Fold change from proteomic results	Consistency
ZP_03275087	0324	9.5/0.32 ↑	↑ +	Y
ZP_03275668	4248	36.2/6.2 ↑	↓ -6.36	N

Note: (1) “**Y**” indicates the transcriptional level and expression of gene is consistency, “N” indicates inconsistency

(2) In “Fold change” column, “+” or “-” indicates the visible or not visible protein spots.

(3) The positive and negative fold indicate the protein spots with >3-fold up-regulation and down-regulation in 15°C and 45°C treatment group vs 35°C.

Finally, we performed metabolic pathway positioning using KEGG analysis, and found 27 genes with >3-fold up-regulated proteins and 5 genes with >3-fold down-regulated proteins. In addition, 18 protein spots were only observed in the 15°C treatment, while they were not visible in the control group. As shown in Figure 1 and Table 1, these genes were involved in energy metabolism (photosynthesis, respiratory electron transport), post-translational modification, protein turnover, chaperones, carbohydrate transport and metabolism, coenzyme metabolism and cell envelope biogenesis. At 45°C, there were 10 genes with >3-fold up-regulated proteins and 2 genes with >3-fold down-regulated proteins. A total of 12 protein spots were only visible in the 45°C treatment and not visible in the control group. These genes were involved in energy metabolism, carbohydrate transport and translation. Moreover, 7 genes for the 15°C treatment and 8 genes for the 45°C treatment were found to have unknown functions, belonging to hypothetical or uncharacterized proteins.

## Discussion

### Sample preparation

In proteome studies, sample preparation is the first key step especially for algal cell samples, which contain many secondary metabolites that may interfere with electrophoresis profiles. 2-DE procedures were optimized using three approaches. Firstly, the proteins were sedimented by TCA/cold acetone, which inhibited protein hydrolysis due to the presence of protease and removed a large proportion of secondary metabolites, such as pigments, quinones and phenols. Secondly, we chose pH 4-7 IPG drystrips, with a narrow pH range, instead of pH 3-10 drystrips, which improved the resolution of protein spots in 2-DE profiles by increasing the loading quantity of the sample and the numbers of protein spots with low abundance. Finally, we utilized the improved protein dissolving buffer to enhance the dissolving efficiency for proteins.

 If biological questions can be answered using quantitative proteomics, it is essential to design experiments which have sufficient power to be able to detect changes in expression. Sample pooling is a strategy that can be used to reduce the variance but still allow studies to encompass biological variation. Underlying sample pooling strategy is the biological averaging assumption that the measurements taken on the pool are equal to the average of the measurements taken on the individuals. Karp et al. reported no evidence of a systematic bias triggered by sample pooling for DIGE and that pooling can be useful in reducing biological variation [26,27]. The previous study also demonstrated an alternative smart pooling strategy in which different samples, not necessarily biological replicates, are pooled in an information theoretic efficient way [28]. Pooling of RNA samples is generally applied to obtain samples that represent the average signal of biological replicates of a single treatment. For toxicogenomics, pooling RNA of samples treated by different compounds could in the same way summarize these compounds to a single sample with average signals per class. Pooling RNA from compounds of a class substantially increased power to detect significantly regulated genes between classes because variability between pooled samples was much lower. Within pools the vast majority of genes maintained patterns of expression compared to the separately hybridized samples, especially in regulated genes [29]. Engel et al. found that the most abundant T-RFLP (terminal restriction fragment polymorphism) peaks were generally shared between biological replicates and pooled samples, and that sample pooling was still effective for the analyses of ecological key players [30]. Especially for homogeneous biological samples, pooling of RNA samples of biological replicates can be generally applied for representing the average information of biological replicates of a single treatment [29]. Therefore, we mixed the extracted protein samples in the three biological replicates into one sample for 2-DE in order to simplify the subsequent 2-DE procedures considering that *Arthrospira* (*Spirulina*) *platensis* was a kind of homogeneous biological material. 

### Target genes and its related mechanisms

 Temperature is an important abiotic factor restricting the growth and distribution of cyanobacteria. The up- or down-regulation of the target genes, as a response to adverse environmental conditions, can lead to the corresponding regulation of their encoded proteins. Thus, we investigated the differentially expressed proteins of ASP at different temperatures by proteomic techniques and identified differentially expressed genes. It can be expected that some differentially expressed genes play an important role in response to abnormal temperatures.

 Some findings suggested that temperature stress for a long time below the freezing point could result in destroyed cell structure, cell cycle current stoppage, solution leakage from cells, and a broken equilibrium state with the inner hormone system. Therefore, the cyanobacteria will experience a series of metabolic obstacles [31]. ASP can tolerate low temperature by increasing the content of intracellular soluble sugar, protein, and free proline to change the metabolism *in vivo* [32]. The effects of high temperature on ASP mainly include direct and indirect damage associated with photosynthesis and respiration metabolic activity, inhibiting biological activity of large molecules, and accumulating toxic substances that lead to oxidative damage of biological macromolecules [33].

#### (1): Heat shock proteins (protein turnover and chaperones)

We found that the seven genes related to protein chaperones polypetide folding and protein turnover in ASP were significantly regulated or newly produced under temperature stresses (Table S3). The Chaperonin Hsp60/GroEL (ORF1976 and ORF1434) is a special protein associated with nascent polypeptide chain folding, oligomeric protein assembly and transmembrane transport [34]. The Hsp60 in mitochondrion mainly participate in protein processing, transport, positioning and assembly. The chloroplast Hsp60 encoded by nuclear genes abundantly exists under normal conditions. However as a response to high temperature, the denatured proteins increase *in vivo*, and Hsp60 binds to the denatured protein to maintain their soluble state. In the presence of Mg^2+^-ATP, Hsp60 can change folding proteins to refold into the active conformation, or degrade the denatured protein [35]. For example, Hsp60 can refold the Rubisco enzyme of cyanobacteria and malate dehydrogenase of mitochondrion *in vitro* [36]. This type of molecular chaperone usually plays an important role in protein migration, translation mechanisms on thylakoid membrane surfaces and improving the fluidity of membranes in order to maintain metabolic activity at low temperatures [22,37]. The up-regulation of the molecular chaperone, Hsp60/GroEL, was also found on exposure to low temperature. We inferred that Hsp60/GroEL was involved in biochemical mechanisms through cells that sense temperature changes and protect ASP from being damaged in response to low temperature.

 Moreover, the functional identification results demonstrated that ORF2155 (ZP_06382427), ORF4632 (ZP_06380688) and ORF4633 (ZP_06380637) were related to phycobilisome linker polypeptides, and their significant changes were regulated by Hsp70 and Hsp40 under high or low temperatures. Both Hsp70 and Hsp40 chaperone families in the cytoplasm or the ER play a critical role in folding and secretion of heterologous proteins. The Hsp70 proteins transiently bind and release polypeptides in an ATP-dependent cycle and the Hsp40 proteins regulate this cycle by modulating the ATPase activity of Hsp70. These chaperones direct nascent polypeptide chains through a series of specialized compartments within the secretory pathway. Only those proteins that have surpassed the proofreading and quality control check points in the pathway are secreted. The partner proteins bind to nascent peptide chains thereby stabilizing and directing them for translocation into the ER [38].

#### (2): Energy metabolism (photosynthesis, respiratory and electron transport)

As summarized in Tables 7 and 8, the expression levels of some genes involved in energy metabolism (photosynthesis, respiratory and electron transport) were significantly changed. For example, Ferredoxin (Fd, ORF2382) was expressed at low temperature, while this was not the case at high temperature.

**Table 7:** The process of metabolism-related differential protein spots at 15°C.

**15°CSpots#**	**ORF**	**Accession NO.**	**Energy metabolism**	**Fold change**	**Name**
137	ORF5159	ZP_03271326	Allophycobilisome, alpha subunit	+	ApcA
140	ORF4634	ZP_03271568	Phycocyanin, alpha subunit	+	CpcA
147	ORF4634	ZP_03271568	Phycocyanin, alpha subunit	+	CpcA
332	ORF2382	ZP_03273564	Ferredoxin	+	/
332	ORF2382	ZP_06380716	Bidirectional hydrogenase complex protein HoxU	+	/
435	ORF4632	ZP_06380688	Phycocyanin-associated rod linker polypeptide	3.26± 0.29	CpcC
436	ORF4632	ZP_06380688	Phycocyanin-associated rod linker polypeptide	4.17± 0.28	CpcC
390	ORF2155	ZP_06382427	Phycobilisome rod-core linker polypeptide	+	CpcG

Note: In “Fold change” column, “+”indicates the solely visible protein spots in 15°C treatment gels.

The positive fold indicates the protein spots with >3-fold up-regulation in 15°C vs 35°C treatment group.

**Table 8:** The process of metabolism-related differential protein spots at 45°C.

**45°C Spots#**	**ORF**	**Accession NO.**	**Energy metabolism**	**Fold change**	**Name**
118	ORF4634	ZP_03271568	Phycocyanin, alpha subunit	+	CpcA
143	ORF4634	ZP_03271568	Phycocyanin, alpha subunit	+	CpcA
70			Phycocyanin, alpha subunit	+	CpcA
564	ORF4633	ZP_06380687	Phycocyanin-associated rod linker polypeptide	3.09± 0.28	CpcC
478	ORF2155	ZP_06382427	Phycobilisome rod-core linker polypeptide	+	CpcG
486	ORF2155	ZP_06382427	Phycobilisome rod-core linker polypeptide	5.39± 0.35	CpcG

Note: In “Fold change” column, “+”indicates the solely visible protein spots in 45°C treatment gels.

The positive fold indicates the protein spots with >3-fold up-regulation in 45°C vs 35°C treatment group.

In the process of photosynthesis, [2Fe-2S]-Fd accepts electrons from the PSI and transfers them to Fd:NADP^+^ oxidoreductase to reduce the NADP^+^ [39], suggesting that Fd plays an important role in PSI. However, Fd (protein spot 332, ORF2382) was suppressed at high temperature, which could result in the significant inhibition of PSI at high temperatures [40].

In addition, a large portion of the differentially expressed protein spots was related to phycobilisome (PBS). PBS is a pigment and protein complex that attaches to the outer surface of thylakoid membranes [41,42]. After the differentially expressed protein spots were characterized with their associated metabolic pathway with KEGG functional annotation, we found four photosynthesis-related proteins ApcA (ORF5159), CpcA (ORF4634), CpcC (ORF4632) and CpcG (ORF2155) at 15°C, and three photosynthesis-related proteins CpcA (ORF4634), CpcC (ORF4633) and CpcG (ORF2155) at 45°C. ApcA (ORF5159) is a part of the allophycocyanin (APC) complex belonging to the core of phycobilisome, CpcA (ORF4634) is an alpha subunit of phycocyanin (PC), and CpcC (ORF4632, ORF4633) and CpcG (ORF2155) are phycobilisome linker polypeptides [42].

It is worthy to note that the expression level of CpcC and CpcG increased by more than 3-fold (especially for CpcG by 5.4-fold at 45°C) when ASP was subjected to low or high temperatures (15 or 45°C). The increase of phycobilisome linker polypeptide CpcC and CpcG made the linker more stable between phycocyanin and allo-phycocyanin. These phenomena would protect phycobilisome from uncoupling under abnormal temperatures. Additionally, the expression level of phycobilisome protein ApcA and the phycocyanin alpha subunit CpcA increased at 15°C, and the level of CpcA increased at 45°C [43,44].

High temperature has a remarkable effect on multiple target sites of thylakoid membranes, which can further affect electronic transfers associated with photosynthesis [41]. The decreasing photochemical activity of PSII results from oxygen-evolving complex inactivation of PSII, structural changes in the thylakoid membranes [45], decreasing of the PSII-mediated electronic transfer rate, and changes of the antenna system conformation by high temperature. On the other hand, cyanobacteria increase the separation between the PBS supermolecules and the thylakoid membrane, which further prompts the disintegration of PBS supermolecules [46]. The expression level of linking polypeptides CpcC and CpcG significantly increased when compared to the control, which demonstrated that ASP took control measures to prevent phycobilisomes from dissociation due to high or low temperatures. Increasing the expression of CpcC and CpcG would lead to increasing stability of phycocyanin and the related stability between PC and APC. In addition, a slight increase of expression level for ApcA and CpcA subunits was observed at 15°C, and a similar case for CpcA subunits was observed at 45°C. The above results imply adaptation mechanisms of ASP to temperature, which helps explain ASP’s survival and growth under abnormal temperatures [47].

Phycobiliproteins are highly variable in abundance under different environmental conditions and very dynamic in their synthesis and turnover or group modification. For most phycobiliproteins, post-translational modifications are catalyzed by enzymes called bilin lyases, and the methylation of a conserved asparagine (Asn) present at beta-72, which occurs on the beta-subunits of all phycobiliproteins [48].These core proteins, such as ApcA, CpcA and CpcG, are active in light harvesting and can not be completely absent from the control (35°C) group. Our experimental results were acquired by screening the differentially expressed proteins according to relevant statistical standards. Perhaps, due to the high variability of phycobiliproteins, they all showed a significantly expressed up-regulation at 45°C and 15°C. On the contrary, the expression of phycobiliproteins at the control temperature (35°C) was so low that they did not fall within the selected statistical criteria range, and thus they were considered as negatively expressed proteins. As for ApcA, CpcA and CpcG, whether or not their location on the 2DE gels changes in the different temperature treatments remains a question for further investigation. Although some of the proteins were not found to be positively expressed, this does not necessarily demonstrate the non-existence of these proteins in the gels.

#### (3): Carbohydrate transport and metabolism

The high up-regulation for ORF5508 (Ribulose-bisphosphate carboxylase, Rubisco), ORF4921 (Phosphoglycerate mutase, PGAM) and ORF4691 (Transketolase) was observed at 15°C. In contrast, ORF4691 (Transketolase), ORF3807 (Glyceraldehyde-3-phosphate dehydrogenase, GAPDH) and ORF2124 (Carbohydrate-selective porin) were the specific proteins up-regulated at 45°C. Transketolase is involved in both the reductive pentose phosphate cycle (RPPC) and the oxidative pentose phosphate cycle (OPPC). Ndong et al. [49] found that transketolase was expressed to a higher level when growing at 5°C compared to 20°C in winter rye. This is consistent with reports that growth and development of wheat, rye and Arabidopsis at low temperatures induce a general increase in the activities and levels of specific enzymes of RPPC and the sucrose biosynthesis pathway. Additionally, it has been proposed that the transketolase reaction may be considered near-limiting in the homeostatic regulation of carbon ﬂux through the RPPC [49]. GAPDH is a key enzyme in the reductive pentose phosphate cycle. The cold adaptation of the lactic acid bacterium *Lactococcus lactis* results in disruption of sugar metabolism. In *Lactobacillus rhamnosus* HN001, GAPDH is not induced upon cold shock, but it is up-regulated when prestressed with heat (50°C) [50]. To date, few studies have examined the correlation of carbohydrate-selective porin at low temperatures.

#### (4): Translation, ribosomal structure and biogenesis

Under the low and high temperature cultivated conditions, there were 6 genes with up-regulated expression: ORF4248 (Cysteinyl-trna synthetase), ORF4125 (Tyrosyl-trna synthetase) and ORF1804 (Hypothetical protein) for the 15°C treatment, and ORF0073 (Ribosomal protein L6), ORF1074 (spot 433, Elongation factor TS) and ORF1074 (spot 514, Elongation factor TS) for the 45°C treatment. The above genes strongly expressed a series of proteins to alleviate the damage of high or low temperatures through participation in new peptide production, transportation, folding, assembly, positioning, refolding and degradation of denatured proteins [51].

#### (5): Coenzyme metabolism

At 15°C, the 5 differentially expressed proteins were involved in 4 up-regulated genes (ORF5578 (UBA/THIF-type NAD/FAD binding protein), ORF4133 (pyridoxal phosphate biosynthetic protein), ORF1829 (radical SAM domain-containing protein) and ORF4139 (tetrahydrofolatede hydrogenase). However, the 4 genes did not express at 45°C. Their preferential expression at low temperature prompted formation of some gene products, which were not expressed in the control, such as the DEAD box RNA helicase (crhR), fatty acid desaturation enzyme, RNA molecular chaperones, RNA binding protein, proline and NADH dehydrogenase subunit [45]. These substances can inhibit ice crystal formation, maintain osmotic balance, antioxidant activity and systematic stability of cell membranes, and thus they can enhance low temperature tolerance in ASP [52].

#### (6): Unassigned Functional genes

Additionally, we detected expression of 7 (15 °C treatment) and 8 genes (45°C treatment) with unassigned functions that were up-regulated at remarkably high levels (Table S3C). These genes with unknown functions may play an important role in resistance to high and low temperatures in ASP. Therefore, it is of great importance to predict the role of these unknown candidate genes induced as a temperature response. The expression of some genes with unknown functions was found to be appreciably down-regulated, such as ORF253, ORF4030 and ORF0361. Therefore, it is necessary to further study the expression regulation mechanisms of these genes at the transcriptional level under abnormal temperature conditions.

#### (7): The other bacterial genes

The 6 proteins not having high homology in ASP (YP_002248469, YP_002485086, ZP_04040440, YP_001999788, YP_912068, ZP_05256482) did have high homology with other prokaryotes, which belonged to *Hermodesulfovibrio yellowstonii, Cyanothece* *sp.* PCC 7425, *Meiothermus ruber DSM 1279, Mycoplasma arthritidis, Chlorobium phaeobacteroides and Bacteroides*, respectively. All of the above prokaryotes were associated with high temperature-tolerant bacteria, with the exception of *Cyanothece* *sp.* PCC 7425. These results suggest that the 6 proteins come from the target algal strains, not from contamination of other bacteria. In addition, gene “transfer or appropriate” phenomenon occurred in the 6 genes due to long-term environmental adaptation. Perhaps these genes play an important role in tolerating abnormal temperatures.

Some ASP genes, showing inconsistency between transcription and translation levels, were not directly associated with heat and cold tolerance, and perhaps their main roles are regulatory effects [53]. Regulation of gene expression can be controlled by structural gene activation, initiation of transcription, post-transcriptional processing and transport, mRNA degradation, translation and post-translational processing and protein degradation. The degradation of mRNA transcripts is a possible reason for the inconsistency between transcription and translation levels. Some inducible gene transcripts can be degraded immediately after translation and even in the course of translation [45]. The inconsistency between transcription and translation levels is influenced by many factors, and thus further investigation is required to elucidate its detailed mechanisms.

Obviously, the temperature-tolerant mechanism of ASP is not solely mediated through up- or down-regulation of one metabolite or one metabolic pathway, but through complex interactions from cross-regulation of many physiological, biochemical and metabolic pathways. In conclusion, we identified several interesting genes of ASP that responded to high or low temperature treatment by proteomic analysis. To elucidate the temperature-tolerant mechanisms in ASP, the data from this study will guide us to further investigate the expression regulation of these target genes.

## Supporting Information

Figure S1 **The differentially expressed protein profiles of 2-DE of ASP-YZ at different culture temperatures.** Note: 1A- at 35°C; 1B- at 15°C; 1C- at 45°C.
(DOC)

Figure S2 **The mass spectrum of identified protein spots.**
(DOC)

Figure S3 **The AGE profiles of total RNA in 15°C, 35°C and 45°C temperature treatments.** Note: Lane 1, 15°C; Lane 2, control; Lane 3, 45°C treatment.
(DOC)

Table S1 **The primers used for amplification of target and 16S rRNA genes.**
(DOC)

Table S2 **Annealing and extension temperatures for amplification of the target and 16S rRNA genes.**
(DOC)

Table S3 **A** The differentially expressed proteins between 15°C and 35°C treatments.
Note: (1) In “Fold change” column, “+”indicates the visible protein spots in the 15°C treatment gels, while not visible in the 35°C control gels. “-” indicates the visible protein spots in the 35°C control gels, while not visible in the 15°C treatment gels.
(2) The positive fold indicates the protein spots with >3-fold up-regulation in the 35°C control group, and the negative fold indicates those with >3-fold down-regulation in the 15°C treatment group.
(3) The filled grayness indicates the other 6 genes have high homology with other microorganisms.
**B** The differentially expressed proteins between 45 °Cand 35 °C treatments.
Note: (1) In “Fold change” column, “+”indicates the visible protein spots in the 45°C treatment gels, while not visible in the 35°C control gels. “-” indicates the visible protein spots in the 35°C control gels, while not visible in the 45°C treatment gels.
(2) The positive fold indicates the protein spots with >3-fold up-regulation in the 45°C treatment group, and the negative fold indicates those with >3-fold down-regulation in the 45°C treatment group.
**C** The differentially expressed proteins between 15°C and 45°C treatments.
Note: (1) In “Fold change” column, “+^15^” indicates the solely visible spots in the 15°C treatment gels, and “+^45^” indicates the solely visible protein spots in the 45°C treatment gels.
(2) The positive fold indicates the protein spots with >3-fold up-regulation in the 15°C treatment when compared to the 45°C treatment.
(DOC)
