# Supplementary material for: Proteomic Analysis and qRT-PCR Verification of Temperature Response to Arthrospira (Spirulina) platensis
Source: PLoS One. 2013 Dec 12;8(12):e83485. doi: 10.1371/journal.pone.0083485 (PMC3861494; doi:10.1371/journal.pone.0083485)
Supplement: Figure S1 — The differentially expressed protein profiles of 2-DE of ASP-YZ at different culture temperatures. Note: 1A- at 35°C; 1B- at 15°C; 1C- at 45°C. (DOC) [file pone.0083485.s001.doc]

**Figure S**1A

Control gel I at 35℃


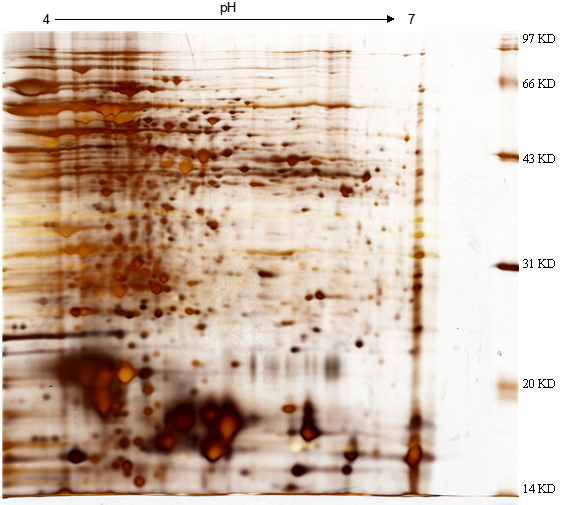


Control gel II at 35℃


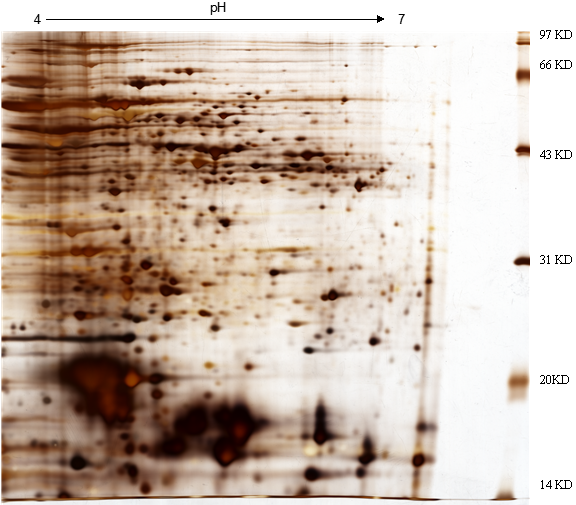


Control gel III at 35℃


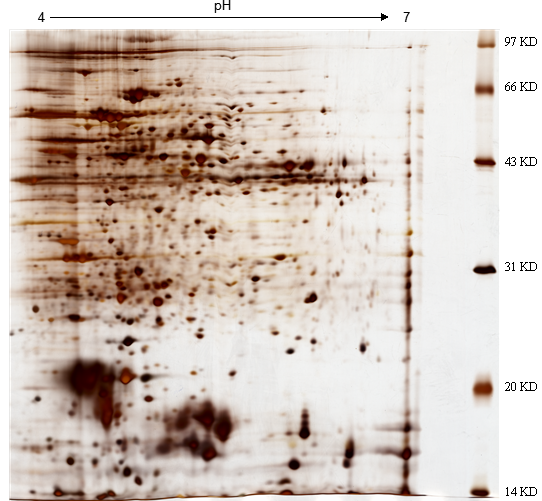


Digit-marking spots gel at 35℃


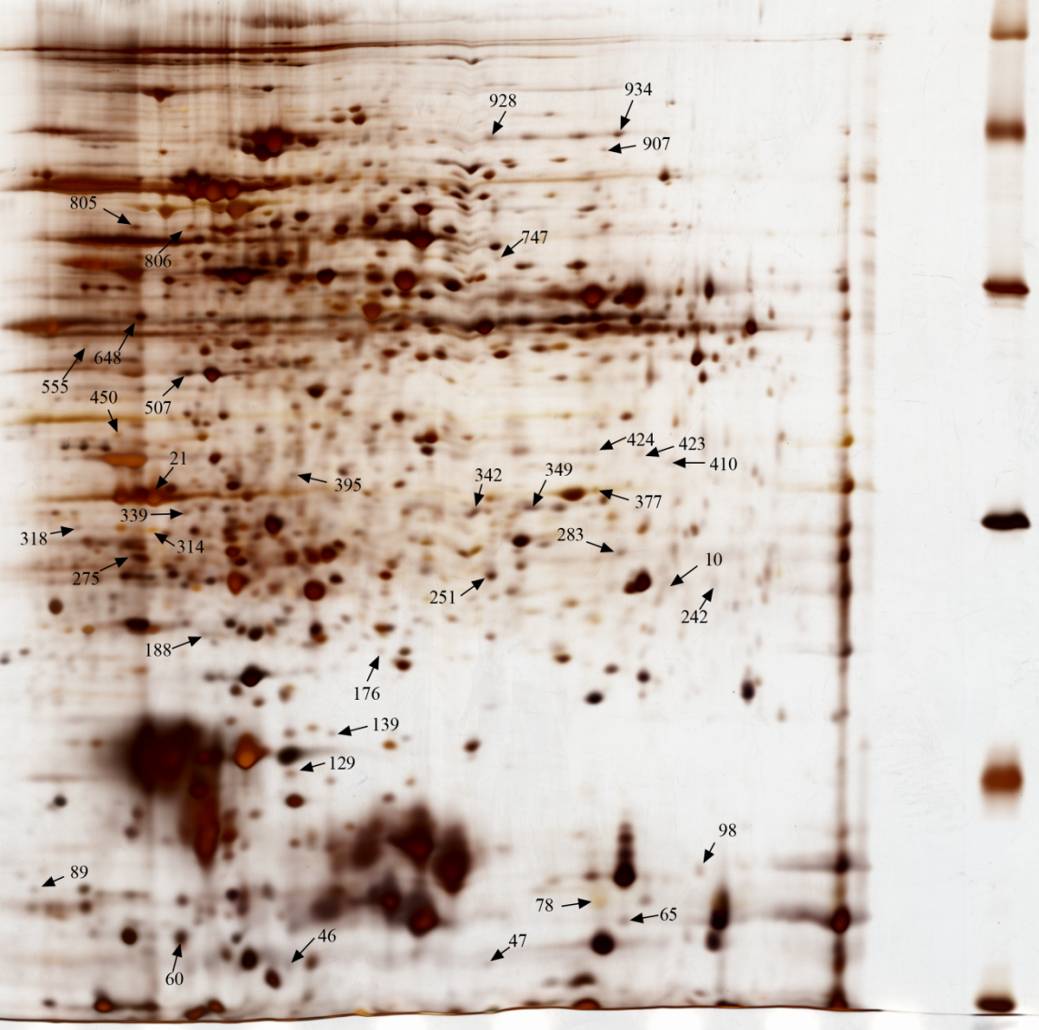


pH 4-7

14kDa

97kDa

20kDa

66kDa

43kDa

31kDa

**Figure S1B**

Treatment gel I at 15℃


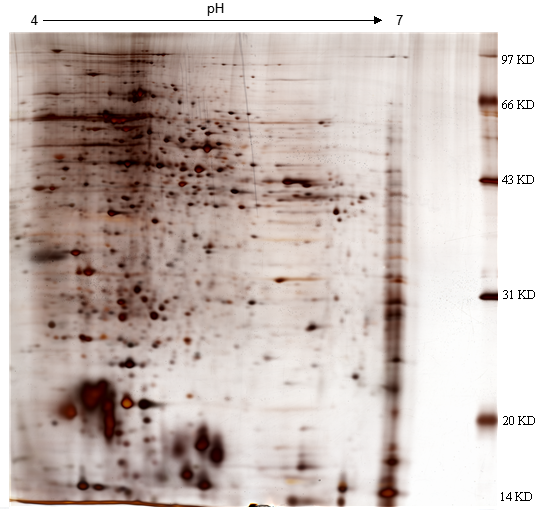


Treatment gel II at 15℃


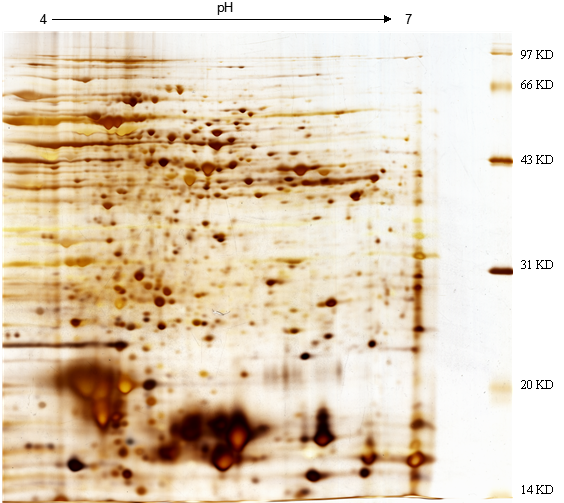


Treatment gel III at 15℃


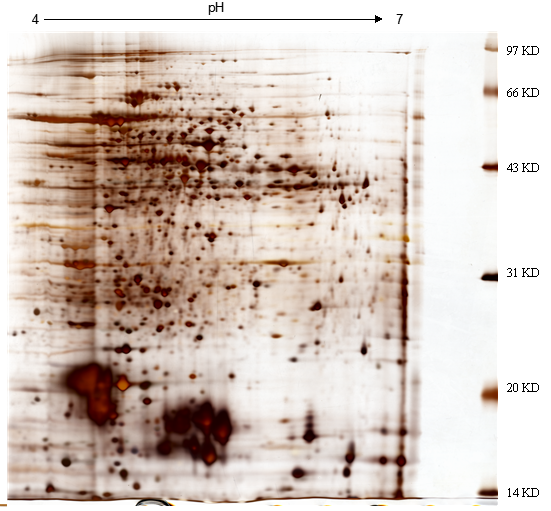


Digit-marking spots gel at 15℃

14kDa

20kDa

97kDa

43kDa

66kDa


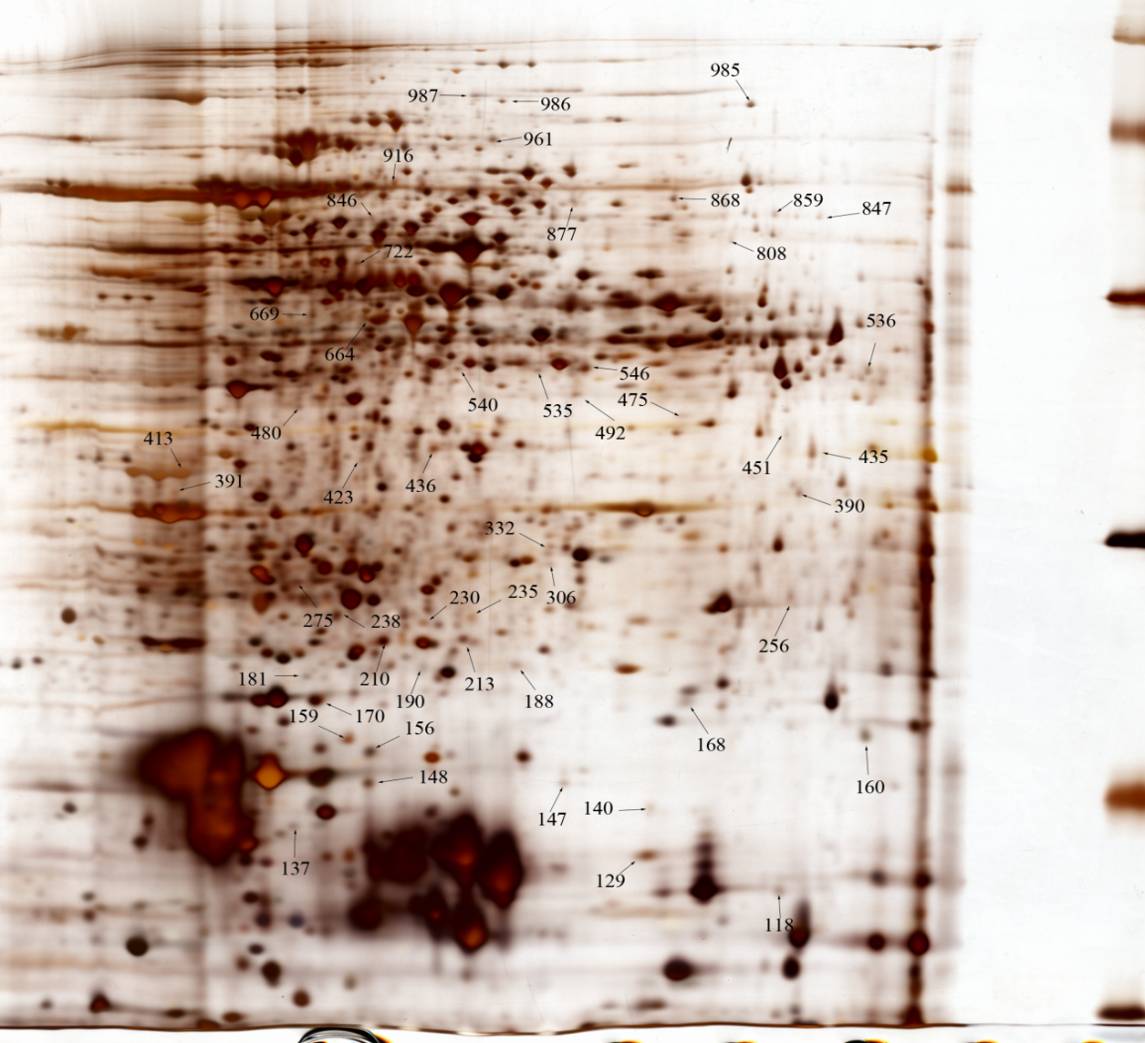


pH 4-7

31kDa

**Figure S1C**

Treatment gel I at 45℃


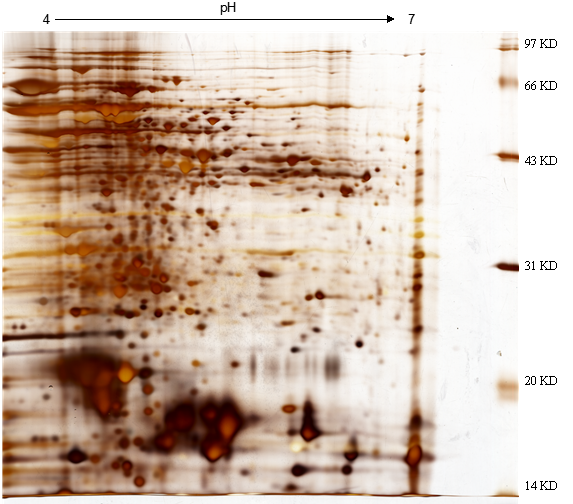


Treatment gel II at 45℃


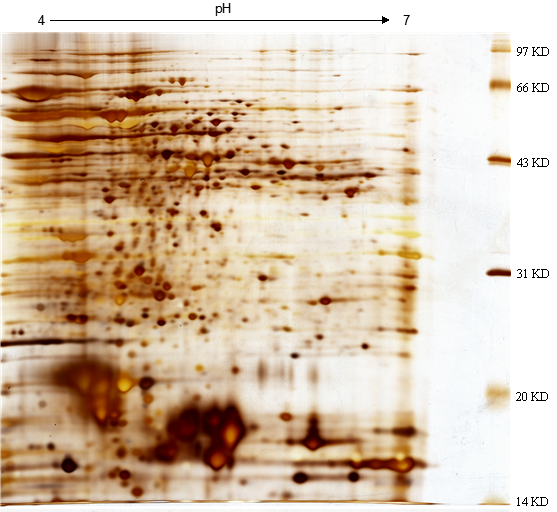


Treatment gel III at 45℃


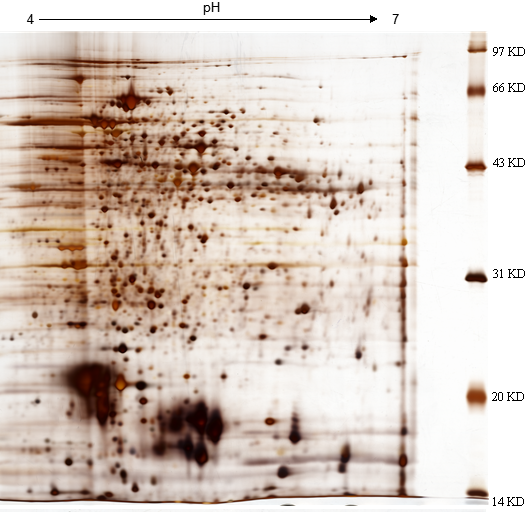


Digit-marking spots gel at 45℃


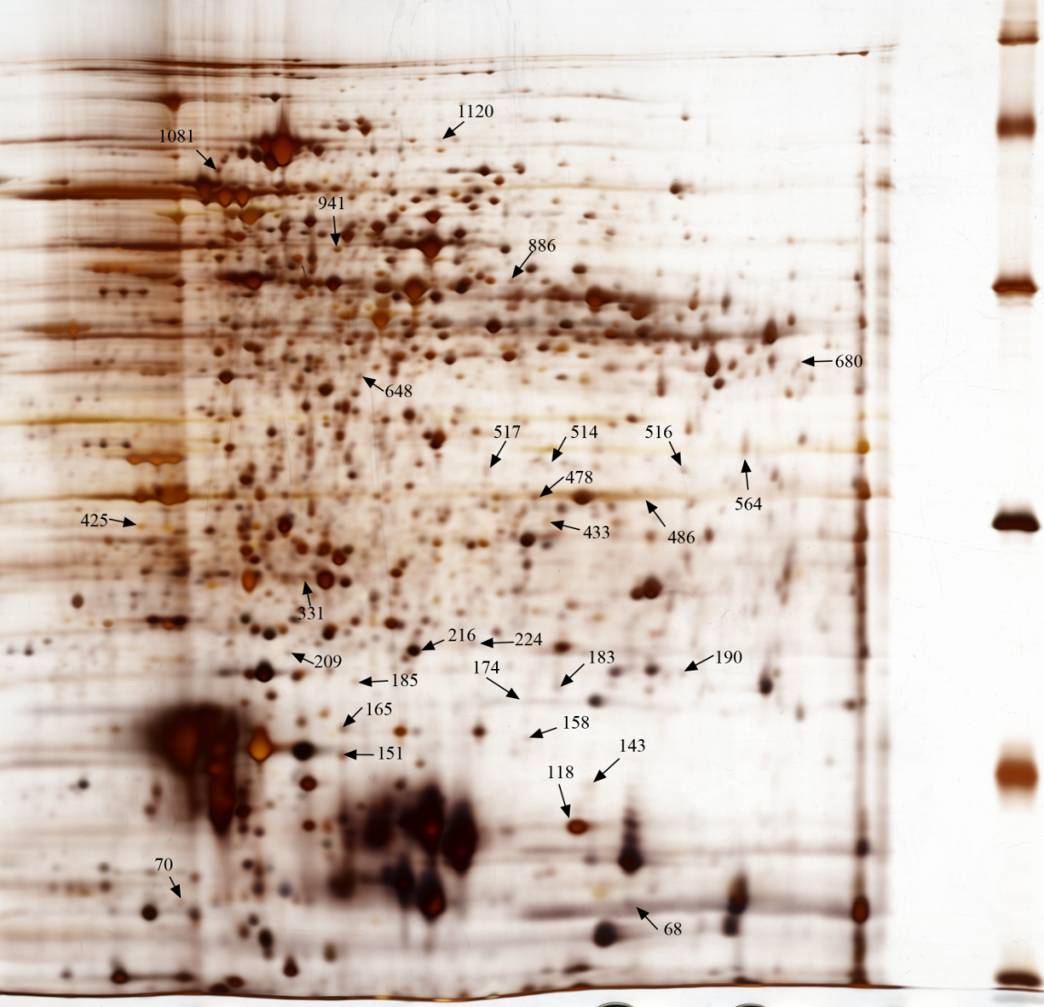


pH 4-7

14kDa

20kDa

43kDa

31kDa

66kDa

97kDa
