# Supplementary figures and images for: Proteomic Analysis and qRT-PCR Verification of Temperature Response to Arthrospira (Spirulina) platensis
Source: PLoS One. 2013 Dec 12;8(12):e83485. doi: 10.1371/journal.pone.0083485 (PMC3861494; doi:10.1371/journal.pone.0083485)

**Figure S2**


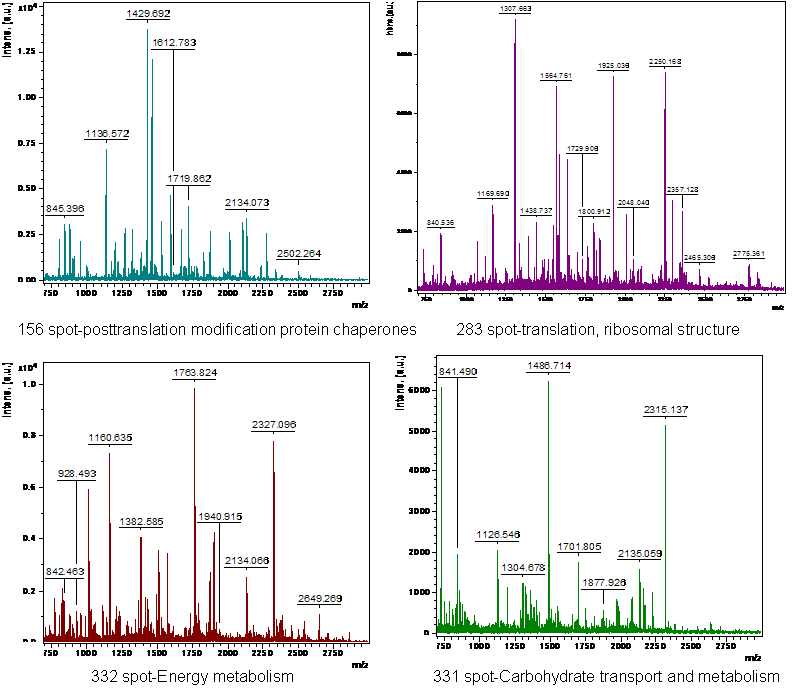

Supplement: Figure S2 — The mass spectrum of identified protein spots. (DOC) [file pone.0083485.s002.doc]

**Figure S3**


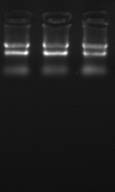


1 2 3

23S

16S

5S

Supplement: Figure S3 — The AGE profiles of total RNA in 15°C, 35°C and 45°C temperature treatments. Note: Lane 1, 15°C; Lane 2, control; Lane 3, 45°C treatment. (DOC) [file pone.0083485.s003.doc]
