# Supplementary material for: Proteomic Analysis and qRT-PCR Verification of Temperature Response to Arthrospira (Spirulina) platensis
Source: PLoS One. 2013 Dec 12;8(12):e83485. doi: 10.1371/journal.pone.0083485 (PMC3861494; doi:10.1371/journal.pone.0083485)
Supplement: Table S1 — The primers used for amplification of target and 16S rRNA genes. (DOC) [file pone.0083485.s004.doc]

**Table S1**

| ORF | Forward primer sequence | Reverse primer sequence | Amplified fragment length |
| --- | --- | --- | --- |
| 4248 | ACGCTTCCGAGTGGCTAT | CTGGTGGTTGTGCTTGTG | 215bp |
| 4632 | GGATTCGGATGGTTGGTC | CTAAATAGGCGTAATGCTG | 247bp |
| 324 | ATTCCTTCCTGGCGACTA | CCGCTGCTGATTATTGAC | 257bp |
| 4935 | ACGGGAGTTGATGTTATTG | CTTAGGTTCAGCGGGTTG | 274bp |
| 3909 | CCCACAACTCGCCTATCA | CTTCCACCAACTTGACCC | 231bp |
| 5578 | CGTAGATGGCACCGATAA | TAAGAGTCCGTCCCTGTC | 287bp |
| 4133 | AAGCCTCAGCCACAGTTA | GATAGCGTGTTTCATTTCC | 287bp |
| 4139 | ATACGAGCCAGGAAGAAC | CGCTAATGGGATTTGATA | 257bp |
| 1368 | CTTGTTCCTCCACCCACC | GCCGAAGTCTGGCTCAAC | 296bp |
| 1976 | AAACCGAAATGAAAGACCG | GTAGCCGACATCAAAGGA | 287bp |
| 2861 | AATGTGATGATGGGTTTG | AGAATAATGCGGCTGTAC | 287bp |
| 4792 | GGGAGGTTATGCTATGGG | GTGGCTGCGTAATGGTTG | 280bp |
| 5692 | CGCCCGAGACTTACAACC | GTGGCAGCATTTCCCTCT | 276bp |
| 1751 | ATTTCCTCCATTGGTAGCG | TTAGCCTGATCCGAGTGC | 269bp |
| 2208 | ATTGCGGAACTGGAAGGG | GCCAACATTGCCAAACTC | 281bp |
| 361 | TGAGTTGACTCCGGTCTATGC | CACCTTCGGCTGCCTGTA | 281bp |
| 5159 | GAGCGCATCATCAAGGAA | AGACAGCAGGGAAGTGGC | 285bp |
| 4634 | TAACCGAAGCAGTTTCTATC | GCACATTTGTCCTTACCG | 241bp |
| 1961 | TCCGCCTGGAACGTAATC | CCCGTGCTAAAGCCTCAA | 285bp |
| 1242 | TGCCTTAGCCTTTGTAGC | TTCGGTTTCTTCCCAGAT | 274bp |
| 1074 | CAGAGGGAATAGTCGGTAG | TTGAGGCGTTTGTCAATA | 292bp |
| 5508 | GCTTTGTTGCCTATCCCT | CACCACGCAGACACTCATA | 291bp |
| 4633 | TCGTCAGGTGATGGGTAA | CAGGAGAATCCAGGTAAGAA | 293bp |
| 2155 | CACTACCACGAGTTTCAATAC | GTCGCTAACCCACCAATA | 240bp |
| 1911 | TATCGTCCGCACTAACTT | ACATCCCTGTCTTCACAA | 231bp |
| 1456 | CCTTAGTTATGCCGATTTG | TGAGGGTCAGACTAGGAAC | 227bp |
| 4030 | GGCAACGAGAACGATACAG | GCCATTGAGCAACCGTAT | 288bp |
| 278 | GGCACAGTGGTAGAAGGT | CTGGTCCAGTGGTTTGAT | 295bp |
| 376 | AATGATCCTTGATGCCTTAT | ATCGGAACCCAGACAGAA | 295bp |
| 73 | ACATGGTGGATGGAGTTTC | TTGCCGACGAGTTCTTTA | 208bp |
| 4691 | GCATTTCCGCAAAGCAGT | CAACCAATTCGGGAAGGA | 245bp |
| 4543 | TGCCAGATAAGTTAGATGCC | GTGGAGTTTCCGCCGTGA | 255bp |
| 1251 | ACTATCACCCGTGGCTTAC | AACCGACTCAATCCAAACAG | 297bp |
| 3656 | AACCTATCCTCATATCCCTG | CCATCTTCCTTATCACCC | 264bp |
| 2281 | CGGAGACTTCCCAACAGC | AGGCAAATCCGACAGAGC | 203bp |
| 1434 | TAGCCTTTGACGAAGAATC | ACCATCTCCAGCCACATC | 248bp |
| 4662 | ATTACCGATGCTATTCAC | TTGTTTCGCCATGCGTTT | 273bp |
| 2593 | AGGCTGAGAAGGTGATAG | TCTTCGTGGACAGTATCG | 217bp |
| 16S rRNA | AAGCCTGACGGAGCAAGA | GGACGCTTTACGCCCAAT | 177bp |
