# Supplementary material for: Proteomic Analysis and qRT-PCR Verification of Temperature Response to Arthrospira (Spirulina) platensis
Source: PLoS One. 2013 Dec 12;8(12):e83485. doi: 10.1371/journal.pone.0083485 (PMC3861494; doi:10.1371/journal.pone.0083485)
Supplement: Table S2 — Annealing and extension temperatures for amplification of the target and 16S rRNA genes. (DOC) [file pone.0083485.s005.doc]

**Table S2**

| ORF and the corresponding internal standard | Annealing T℃ | Standard curve | Regression coefficient (R^2^) | Amplified efficiency% |
| --- | --- | --- | --- | --- |
| 4632，324，4935，4133，1976，5508，278，4691，5159，4633，1456，376，2593，3656，2155，5578，2208，4634，4030，1434 (16S rRNA^1^) | 52℃ | y = -3.354x + 45.172 | 0.994 | 98.7 |
| 4248，5692，3909，1751，4792 ，1961，2281(16S rRNA^2^) | 54℃ | y = -3.878x + 50.014 | 0.994 | 81.1 |
| 1368，361，4543，1251(16S rRNA^3^) | 56℃ | y = -3.18x + 42.666 | 0.998 | 106.3 |
| 1911，1242，73 (16S rRNA^4^) | 50℃ | y = -3.44x + 47.638 | 0.995 | 95.3 |
| 4139，2861，1074，4662(16S rRNA^5^) | 48℃ | y = -2.968x + 41.617 | 0.982 | 117.2 |
