# Supplementary material for: Proteomic Analysis and qRT-PCR Verification of Temperature Response to Arthrospira (Spirulina) platensis
Source: PLoS One. 2013 Dec 12;8(12):e83485. doi: 10.1371/journal.pone.0083485 (PMC3861494; doi:10.1371/journal.pone.0083485)
Supplement: Table S3 — A The differentially expressed proteins between 15°C and 35°C treatments. Note: (1) In “Fold change” column, “+”indicates the visible protein spots in the 15°C treatment gels, while not visible in the 35°C control gels. “-” indicates the visible protein spots in the 35°C control gels, while not visible in the 15°C treatment gels. (2) The positive fold indicates the protein spots with >3-fold up-regulation in the 35°C control group, and the negative fold indicates those with >3-fold down-regulation in the 15°C treatment group. (3) The filled grayness indicates the other 6 genes have high homology with other microorganisms. B The differentially expressed proteins between 45 °Cand 35 °C treatments. Note: (1) In “Fold change” column, “+”indicates the visible protein spots in the 45°C treatment gels, while not visible in the 35°C control gels. “-” indicates the visible protein spots in the 35°C control gels, while not visible in the 45°C treatment gels. (2) The positive fold indicates the protein spots with >3-fold up-regulation in the 45°C treatment group, and the negative fold indicates those with >3-fold down-regulation in the 45°C treatment group. C The differentially expressed proteins between 15°C and 45°C treatments. Note: (1) In “Fold change” column, “+15” indicates the solely visible spots in the 15°C treatment gels, and “+45” indicates the solely visible protein spots in the 45°C treatment gels. (2) The positive fold indicates the protein spots with >3-fold up-regulation in the 15°C treatment when compared to the 45°C treatment. (DOC) [file pone.0083485.s006.doc]

**Table S3A**

| **Spot#** | **Accession NO.** | **ORF** | **Gene product** | **Theor./** | **Exper.** | **Matched Peptides** | **Cov** | **Score** | **E-value** | **Fold change**  (Mean± SD) |
| --- | --- | --- | --- | --- | --- | --- | --- | --- | --- | --- |
|  |  |  |  | **MW(kDa)** | **pI** |  |  |  |  |  |
| **Amino acid transport and metabolism** | | | | | | | | | | |
| 160 | ZP_06381012 | ORF4935 | Acetolactate synthase 3 regulatory subunit [*Arthrospira platensis*] | 19.26/22.13 | 6.62/6.80 | 14 | 64% | 154 | 4.20E-10 | 3.10±0.22 |
| 492 | ZP_06381042 | ORF1242 | Cysteine synthase A [*Arthrospira platensis*] | 34.84/37.37 | 5.33/5.85 | 23 | 67% | 151 | 8.30E-10 | 3.39±0.15 |
| 535 | ZP_06382239 | ORF3909 | Aspartate-semialdehyde dehydrogenase [*Arthrospira platensis*] | 37.27/38.57 | 5.38/5.72 | 22 | 64% | 209 | 1.30E-15 | 3.43±0.29 |
| 395 | ZP_06380542 | ORF0089 | Diaminopimelate epimerase [*Arthrospira platensis*] | 31.57/32.88 | 4.97/5.04 | 9 | 52% | 94 | 4.00E-04 | - |
| **Carbohydrate transport and metabolism** | | | | | | | | | | |
| 846 | ZP_03272404 | ORF5508 | Ribulose-bisphosphate carboxylase [*Arthrospira maxima*] | 53.91/53.08 | 6.04/5.19 | 32 | 50% | 228 | 1.70E-17 | + |
| 648 | ZP_06380758 | ORF3656 | Fructose-1,6-bisphosphate aldolase [*Arthrospira platensis*] | 38.99/41.19 | 5.60/4.49 | 13 | 33% | 168 | 1.60E-11 | -4.39±0.33 |
| 546 | ZP_06381579 | ORF3807 | Glyceraldehyde-3-phosphate dehydrogenase, type I [*Arthrospira platensis*] | 36.89/39.08 | 5.41/5.90 | 15 | 44% | 148 | 1.70E-09 | + |
| 847 | ZP_06381851 | ORF4921 | Phosphoglycerate mutase [*Arthrospira platensis*] | 50.68/53.08 | 6.01/6.68 | 14 | 31% | 107 | 2.10E-05 | + |
| 985 | ZP_06382051 | ORF4691 | Transketolase [*Arthrospira platensis*] | 72.98/73.83 | 5.78/6.41 | 34 | 60% | 216 | 2.60E-16 | 3.13±0.18 |
| 46 | ZP_06382410 | ORF4142 | Phosphoglycerate kinase [*Arthrospira platensis*] | 42.36/14.81 | 4.92/5.03 | 6 | 25% | 65 | 3.30E-01 | - |
| 450 | ZP_06382037 | ORF4701 | Pfkb [*Arthrospira platensis*] | 36.86/34.83 | 4.61/4.41 | 8 | 38% | 82 | 7.30E-03 | - |
| 747 | ZP_03272404 | ORF5508 | Ribulose-bisphosphate carboxylase [*Arthrospira maxima*] | 53.91/46.33 | 6.04/5.78 | 25 | 42% | 230 | 1.00E-17 | - |
| 907 | ZP_06382051 | ORF4691 | Transketolase [*Arthrospira platensis*] | 72.98/62.67 | 5.78/6.16 | 14 | 36% | 148 | 1.60E-09 | - |
| 928 | ZP_06382051 | ORF4691 | Transketolase [*Arthrospira platensis*] | 72.98/64.76 | 5.78/5.75 | 21 | 45% | 204 | 4.10E-15 | - |
| **Coenzyme metabolism** | | | | | | | | | | |
| 664 | ZP_03272326 | ORF5578 | UBA/THIF-type NAD/FAD binding protein [*Arthrospira maxima*] | 43.01/41.67 | 5.06/5.20 | 15 | 49% | 147 | 2.10E-09 | 3.75±0.28 |
| 235 | ZP_03275242 | ORF4133 | Pyridoxal phosphate biosynthetic protein pdxj [*Arthrospira maxima*] | 26.10/27.02 | 5.30/5.52 | 7 | 47% | 114 | 4.20E-06 | 4.69±0.43 |
| 536 | ZP_06381750 | ORF4139 | Tetrahydrofolatede hydrogenase/cyclohydrolase [*Arthrospira platensis*] | 30.92/38.66 | 6.01/6.79 | 10 | 32% | 76 | 2.50E-02 | 5.49±0.42 |
| **Post-translational modification, protein turnover, chaperones** | | | | | | | | | | |
| 306 | YP_002248469 | ORF3841 | Metalloprotease ftsh [*Thermodesulfovibrio yellowstonii*] | 66.90/30.37 | 6.12/5.77 | 15 | 26% | 89 | 1.30E-03 | + |
| 156 | ZP_03273475 | ORF1368 | Redoxin domain protein [*Arthrospira maxima*] | 19.78/21.51 | 4.88/5.18 | 15 | 66% | 133 | 5.20E-08 | 5.85±0.43 |
| 159 | ZP_03273475 | ORF1368 | Redoxin domain protein [*Arthrospira maxima*] | 19.78/21.71 | 4.88/5.11 | 19 | 68% | 158 | 1.70E-10 | 3.10±0.22 |
| 916 | ZP_03275215 | ORF1976 | Chaperonin GroEL [*Arthrospira maxima*] | 58.21/57.65 | 5.00/5.25 | 28 | 56% | 183 | 5.20E-13 | 3.84±0.29 |
| 159 | ZP_06384587 | ORF1368 | Peroxiredoxin, putative [*Arthrospira platensis*] | 19.78/21.71 | 4.87/5.11 | 17 | 64% | 141 | 8.30E-09 | 5.85±0.35 |
| 156 | ZP_06384587 | ORF1368 | Peroxiredoxin, putative[*Arthrospira platensis*] | 19.78/21.51 | 4.87/5.18 | 13 | 62% | 117 | 2.10E-06 | 3.45±0.26 |
| 139 | ZP_03273475 | ORF1368 | Redoxin domain protein [*Arthrospira maxima*] | 19.78/21.51 | 4.88/5.18 | 13 | 59% | 125 | 3.30E-07 | 5.85±0.49 |
| 275 | ZP_06380867 | ORF2281 | Peptidyl-prolyl cis-trans isomerase, cyclophilin type [*Arthrospira platensis*] | 23.92/28.93 | 4.74/4.49 | 6 | 36% | 75 | 3.60E-02 | -3.12±0.28 |
| 60 | ZP_03273444 | ORF3231 | Alkyl hydroperoxide reductase/ Thiol specific antioxidant/ Mal allergen [*Arthrospira maxima*] | 15.69/15.45 | 4.66/4.63 | 5 | 42% | 84 | 3.70E-03 | - |
| 805 | ZP_06380920 | ORF1434 | ChaperoninGroEL [*Arthrospira platensis*] | 57.43/50.62 | 5.00/4.47 | 24 | 50% | 271 | 8.20E-22 | - |
| 806 | ZP_06380920 | ORF1434 | Chaperonin GroEL [*Arthrospira platensis*] | 57.43/50.62 | 5.00/4.67 | 26 | 53% | 247 | 2.10E-19 | - |
| **Signal transduction mechanisms** | | | | | | | | | | |
| 475 | ZP_06381964 | ORF0151 | Hypothetical protein aplap_09815 [*Arthrospira platensis*] | 9.72 /6.71 | 7.93/6.23 | 9 | 82% | 96 | 2.50E-04 | + |
| 210 | ZP_06382415 | ORF2861 | Stress protein [*Arthrospira platensis*] | 22.20/26.12 | 4.93/5.23 | 14 | 78% | 111 | 8.30E-06 | 3.82±0.19 |
| 451 | ZP_06383067 | ORF0151 | Hypothetical protein aplap_15418 [*Arthrospira platensis*] | 21.60/35.82 | 5.30/6.57 | 14 | 57% | 99 | 1.40E-04 | + |
| 555 | YP_002485086 | ORF1869 | Multi-sensor signal transduction histidine kinase [*Cyanothece sp.* PCC 7425] | 85.54/39.52 | 5.95/4.29 | 11 | 12% | 74 | 4.40E-02 | - |
| **Transcription / Signal transduction mechanisms** | | | | | | | | | | |
| 480 | ZP_06381260 | ORF4554 | Phage shock protein A, pspa [*Arthrospira platensis*] | 28.18/37.07 | 5.02/4.94 | 10 | 49% | 90 | 9.30E-04 | + |
| **Translation, ribosomal structure and biogenesis** | | | | | | | | | | |
| 859 | ZP_03275668 | ORF4248 | Cysteinyl-trna synthetase [*Arthrospira maxima*] | 55.01/53.51 | 5.87/6.50 | 19 | 38% | 76 | 2.80E-02 | 6.36±0.41 |
| 148 | ZP_04040440 | ORF4125 | Tyrosyl-trna synthetase [*Meiothermus ruber DSM 1279*] | 47.79/19.87 | 5.84/5.17 | 13 | 29% | 76 | 3.00E-02 | 5.02±0.36 |
| 190 | ZP_06382972 | ORF1804 | Hypothetical protein aplap_14933 [*Arthrospira platensis*] | 21.94/24.72 | 5.31/5.35 | 6 | 28% | 79 | 1.30E-02 | 4.62±0.24 |
| 21 | ZP_06384062 | ORF0361 | Peptidase S8 and S53 subtilisin kexin sedolisin [*Arthrospira platensis*] | 44.24/31.74 | 4.59/4.56 | 21 | 47% | 195 | 3.30E-14 | -4.42±0.45 |
| 339 | ZP_06384062 | ORF0361 | Peptidase S8 and S53 subtilisin kexin sedolisin [*Arthrospira platensis*] | 44.24/31.22 | 4.59/4.65 | 16 | 44% | 200 | 1.00E-14 | 3.03±0.27 |
| 283 | ZP_03271593 | ORF4662 | Sigma 54 modulation protein/ribosomal protein S30EA [*Arthrospira maxima*] | 23.97/29.22 | 7.03/6.22 | 9 | 58% | 94 | 4.00E-04 | - |
| 78 | ZP_06384870 | ORF4030 | Hypothetical protein aplap_24737 [*Arthrospira platensis*] | 17.32/16.31 | 5.96/6.15 | 19 | 86% | 254 | 4.10E-20 | - |
| 89 | ZP_06382587 | / | Hypothetical protein aplap_12998 [*Arthrospira platensis*] | 19.30/16.69 | 9.47/4.11 | 14 | 72% | 187 | 2.10E-13 | - |
| 314 | ZP_06384062 | ORF0361 | Peptidase S8 and S53 subtilisin kexin sedolisin [*Arthrospira platensis*] | 44.24/30.34 | 4.59/4.53 | 16 | 45% | 153 | 5.20E-10 | - |
| 423 | ABV01983 | ORF4633 | Cpch [*Arthrospira platensis*] | 30.85/33.93 | 7.82/6.29 | 25 | 60% | 295 | 3.30E-24 | - |
| 424 | ZP_03272395 | ORF5516 | Conserved hypothetical protein [*Arthrospira maxima*] | 41.12/34.02 | 6.04/6.08 | 13 | 50% | 205 | 3.30E-15 | - |
| **Energy metabolism (photosynthesis, respiratory electron transport)** | | | | | | | | | | |
| 390 | ZP_06382427 | ORF2155 | Phycobilisome linker polypeptide [*Arthrospira platensis*] | 29.45/33.10 | 9.25/6.61 | 31 | 76% | 313 | 5.20E-26 | + |
| 137 | ZP_03271326 | ORF5159 | Phycobilisome protein [*Arthrospira maxima*] | 17.44/19.04 | 4.89/4.93 | 16 | 62% | 148 | 1.70E-09 | + |
| 140 | ZP_03271568 | ORF4634 | Phycocyanin, alpha subunit [*Arthrospira maxima*] | 17.70/19.59 | 5.82/6.11 | 11 | 75% | 91 | 8.10E-04 | + |
| 435 | ZP_06380688 | ORF4632 | Phycobilisome linker polypeptide [*Arthrospira platensis*] | 32.78/34.48 | 8.33/6.60 | 18 | 48% | 129 | 1.30E-07 | 3.26±0.29 |
| 436 | ZP_06380688 | ORF4632 | Phycobilisome linker polypeptide [*Arthrospira platensis*] | 32.78/34.72 | 8.33/5.38 | 18 | 48% | 129 | 1.30E-07 | 4.17±0.28 |
| 147 | ZP_03271568 | ORF4634 | Phycocyanin, alpha subunit [*Arthrospira maxima*] | 17.70/20.47 | 5.82/5.83 | 12 | 75% | 81 | 7.60E-03 | + |
| 342 | ZP_06382427 | ORF2155 | Phycobilisome linker polypeptide [*Arthrospira platensis*] | 29.45/31.20 | 9.25/5.69 | 15 | 55% | 218 | 1.60E-16 | -3.15±0.27 |
| 349 | ZP_06382427 | ORF2155 | Phycobilisome linker polypeptide [*Arthrospira platensis*] | 29.45/31.44 | 9.25/5.89 | 25 | 67% | 295 | 3.30E-24 | -4.43±0.36 |
| 47 | ZP_03271327 | ORF4103 | Allophycocyanin, beta subunit [*Arthrospira maxima*] | 17.43/14.81 | 6.26/5.74 | 14 | 84% | 165 | 3.30E-11 | - |
| 318 | ZP_06382427 | ORF2155 | Phycobilisome linker polypeptide [*Arthrospira platensis*] | 29.45/30.46 | 9.25/4.26 | 15 | 53% | 160 | 1.00E-10 | - |
| 410 | ZP_06382427 | ORF2155 | Phycobilisome linker polypeptide [*Arthrospira platensis*] | 29.45/33.46 | 9.25/6.41 | 15 | 54% | 186 | 2.60E-13 | - |
| **Inorganic ion transport and metabolism** | | | | | | | | | | |
| 168 | ZP_06382815 | ORF4792 | DNA starvation/stationary phase protection protein Dps [*Arthrospira platensis*] | 19.67/23.51 | 4.93/6.24 | 10 | 70% | 81 | 8.50E-03 | + |
| 170 | ZP_06382815 | ORF4792 | DNA starvation/stationary phase protection protein Dps [*Arthrospira platensis*] | 19.67/23.83 | 4.93/5.04 | 13 | 70% | 110 | 8.30E-06 | 3.07±0.27 |
| **Intracellular trafficking and secretion** | | | | | | | | | | |
| 423 | YP_001999788 | ORF2422 | Preprotein translocase subunit seca [*Mycoplasma arthritidis*] | 99.38/34.57 | 5.10/5.15 | 21 | 28% | 76 | 2.60E-02 | + |
| **Nucleotide transport and metabolism** | | | | | | | | | | |
| 176 | ZP_03275636 | / | Adenylate kinase [*Arthrospira maxima*] | 21.86/24.72 | 5.22/5.35 | 11 | 65% | 156 | 2.60E-10 | 4.62±0.39 |
| **General function prediction only** | | | | | | | | | | |
| 540 | ZP_06381727 | ORF2131 | Beta-lactamase-like protein [*Arthrospira platensis*] | 34.13/39.06 | 5.12/5.47 | 14 | 40% | 148 | 1.70E-09 | + |
| 10 | ZP_06383388 | ORF2597 | Short-chain dehydrogenase/reductase SDR [*Arthrospira platensis*] | 19.51/27.67 | 5.54/6.38 | 12 | 64% | 129 | 1.30E-07 | - |
| **Cell envelope biogenesis, outer membrane** | | | | | | | | | | |
| 868 | ZP_06383693 | ORF5692 | Phosphateuridyltransferase/glucosamine phosphateacetyl transferase [*Arthrospira platensis*] | 50.14/54.88 | 5.74/6.18 | 23 | 50% | 235 | 3.30E-18 | 3.38±0.27 |
| 213 | ZP_06381424 | ORF1751 | NAD-dependent epimerase/dehydratase [*Arthrospira platensis*] | 23.63/26.06 | 5.15/5.49 | 23 | 78% | 231 | 8.30E-18 | 3.19±0.28 |
| 230 | ZP_06381424 | ORF1751 | NAD-dependent epimerase/dehydratase [*Arthrospira platensis*] | 23.63/26.47 | 5.15/5.36 | 14 | 61% | 174 | 4.20E-12 | 4.40±0.38 |
| 251 | ZP_06381424 | ORF1751 | NAD-dependent epimerase/dehydratase [*Arthrospira platensis*] | 23.63/28.08 | 5.15/5.75 | 12 | 70% | 184 | 4.10E-13 | - |
| **Function unknown** | | | | | | | | | | |
| 188 | ZP_06380822 | ORF1456 | Pentapeptide repeat-containing protein [*Arthrospira platensis*] | 19.86/25.09 | 5.13/5.67 | 16 | 58% | 117 | 2.10E-06 | + |
| 181 | YP_912068 | / | Hypothetical protein Cpha266_1623 [*Chlorobium phaeobacteroides*] | 31.01/24.60 | 6.56/4.97 | 12 | 43% | 84 | 3.90E-03 | + |
| 391 | ZP_03271502 | ORF4558 | Conserved hypothetical protein [*Arthrospira maxima*] | 27.65/33.16 | 4.75/4.53 | 11 | 49% | 88 | 1.80E-03 | + |
| 275 | ZP_06380972 | ORF1961 | Ppic-type peptidyl-prolyl cis-trans isomerase [*Arthrospira platensis*] | 31.57/28.94 | 5.27/4.94 | 22 | 74% | 190 | 1.00E-13 | 3.12±0.24 |
| 961 | ZP_06383545 | ORF2208 | Hypothetical protein aplap_17874 [*Arthrospira platensis*] | 18.76/65.83 | 5.42/5.58 | 13 | 77% | 94 | 4.30E-04 | 4.10±0.28 |
| 413 | ZP_06384062 | ORF0361 | Peptidase S8 and S53 subtilisin kexin sedolisin [*Arthrospira platensis*] | 44.24/34.26 | 4.59/4.57 | 22 | 49% | 166 | 2.60E-11 | 5.25±0.48 |
| 129 | ZP_06384870 | ORF4030 | Hypothetical protein aplap_24737 [*Arthrospira platensis*] | 17.32/18.22 | 5.96/6.07 | 23 | 93% | 266 | 2.60E-21 | 5.02±0.31 |

**Table S3B**

| **Spot#** | **Accession NO.** | **ORF** | **Gene product** | **Theor./** | **Exper.** | **Matched Peptides** | **Cov** | **Score** | **E-value** | **Fold Change**  (Mean± SD) |
| --- | --- | --- | --- | --- | --- | --- | --- | --- | --- | --- |
|  |  |  |  | **MW(kDa)** | **pI** |  |  |  |  |  |
| **Amino acid transport and metabolism** | | | | | | | | | | |
| 648 | ZP_03273305 | ORF0278 | Pyridoxal-5'-phosphate-dependent protein beta subunit [*Arthrospira maxima*] | 34.84/37.95 | 5.28/5.23 | 26 | 69% | 241 | 8.30E-19 | + |
| **Carbohydrate transport and metabolism** | | | | | | | | | | |
| 680 | ZP_03274253 | ORF3807 | Glyceraldehyde-3-phosphate dehydrogenase, type I [*Arthrospira maxima*] | 36.53/38.66 | 6.07/6.79 | 14 | 47% | 108 | 1.70E-05 | 3.19±0.28 |
| 1081 | AAN05011 | ORF5508 | Ribulose-1,5-bisphosphate carboxylase/oxygenase large subunit [*Arthrospira platensis*] | 46.70/60.18 | 6.12/4.73 | 16 | 40% | 75 | 3.30E-02 | 3.39±0.26 |
| 331 | ZP_06381552 | ORF0785 | Ribose-5-phosphate isomerase A [*Arthrospira platensis*] | 25.13/28.28 | 4.96/5.04 | 10 | 46% | 105 | 3.30E-05 | + |
| 1120 | ZP_06381999 | ORF0376 | Transketolase domain protein [*Arthrospira platensis*] | 69.60/64.11 | 5.19/5.52 | 41 | 64% | 297 | 2.10E-24 | + |
| 928 | ZP_06382051 | ORF4691 | Transketolase  [*Arthrospira platensis*] | 72.98/64.76 | 5.78/5.75 | 21 | 45% | 204 | 4.10E-15 | -10.34±0.83 |
| **Post-translational modification, protein turnover, chaperones** | | | | | | | | | | |
| 139 | ZP_03273475 | ORF1368 | Redoxin domain protein [*Arthrospira maxima*] | 19.78/21.51 | 4.88/5.18 | 13 | 59% | 125 | 3.30E-07 | 3.32±0.18 |
| 129 | ZP_03273475 | ORF1368 | Redoxin domain protein [*Arthrospira maxima*] | 19.78/20.11 | 4.88/5.03 | 10 | 49% | 119 | 1.30E-06 | - |
| **Translation, ribosomal structure and biogenesis** | | | | | | | | | | |
| 224 | ZP_03275641 | ORF0073 | Ribosomal protein L6 [*Arthrospira maxima*] | 19.69/25.13 | 10.25/5.63 | 11 | 40% | 104 | 4.20E-05 | + |
| 433 | ZP_06380559 | ORF1074 | Elongation factor TS [*Arthrospira platensis*] | 24.63/31.08 | 5.74/5.90 | 21 | 77% | 155 | 3.30E-10 | + |
| 283 | ZP_03271593 | ORF4662 | Sigma54 modulation protein/ribosomal protein S30EA [*Arthrospira maxima*] | 23.97/29.22 | 7.03/6.22 | 9 | 58% | 94 | 4.00E-04 | - |
| 377 | ZP_06380559 | ORF1074 | Elongation factor TS [*Arthrospira platensis*] | 24.63/32.19 | 5.74/6.13 | 7 | 53% | 75 | 3.40E-02 | - |
| **Energy production and conversion** | | | | | | | | | | |
| 118 | ZP_03271568 | ORF4634 | Phycocyanin, alpha subunit [*Arthrospira maxima*] | 17.70/18.27 | 5.82/6.00 | 11 | 74% | 120 | 1.00E-06 | + |
| 143 | ZP_03271568 | ORF4634 | Phycocyanin, alpha subunit [*Arthrospira maxima*] | 17.70/19.60 | 5.82/6.05 | 14 | 86% | 114 | 4.20E-06 | + |
| 564 | ZP_06380687 | ORF4633 | Phycobilisome linker polypeptide [*Arthrospira platensis*] | 30.87/34.48 | 7.82/6.60 | 30 | 71% | 338 | 1.70E-28 | 3.09±0.28 |
| 486 | ZP_06382427 | ORF2155 | Phycobilisome linker polypeptide [*Arthrospira platensis*] | 29.45/32.42 | 9.25/6.25 | 25 | 70% | 237 | 2.10E-18 | 5.39±0.35 |
| 70 |  | / | Phycocyanin, alpha subunit [*Arthrospira maxima*] | 17.70/16.05 | 5.82/4.60 | 10 | 74% | 98 | 1.70E-05 | + |
| 517 | ABB84420 | ORF4632 | Cpci [*Arthrospira platensis*] | 32.79/33.33 | 8.33/5.68 | 17 | 51% | 127 | 2.10E-07 | + |
| 47 | ZP_03271327 | ORF4103 | Allophycocyanin, beta subunit [*Arthrospira maxima*] | 17.43/14.81 | 6.26/5.74 | 14 | 84% | 165 | 3.30E-11 | - |
| **Cell division and chromosome partitioning** | | | | | | | | | | |
| 507 | ZP_06383180 | ORF4543 | Hypothetical protein aplap15993 [*Arthrospira platensis*] | 38.08/38.00 | 4.59/4.65 | 21 | 58% | 190 | 1.00E-13 | -5.07±0.43 |
| **General function prediction only** | | | | | | | | | | |
| 185 | ZP_06383806 | ORF0728 | Hypothetical protein aplap_19246 [*Arthrospira platensis*] | 22.95/23.52 | 4.99/5.22 | 23 | 70% | 166 | 2.60E-11 | + |
| 886 | ZP_06384147 | ORF2097 | Von Willebrand factor, type A [*Arthrospira platensis*] | 37.19/43.74 | 5.81/5.77 | 19 | 57% | 167 | 2.10E-11 | + |
| 934 | ZP_06381540 | ORF1251 | Hypothetical protein aplap_07632 [*Arthrospira platensis*] | 67.41/65.34 | 5.66/6.21 | 19 | 42% | 166 | 2.60E-11 | - |
| **Inorganic ion transport and metabolism** | | | | | | | | | | |
| 165 | ZP_06383116 | ORF1911 | Adenylylsulfate kinase [*Arthrospira platensis*] | 19.90/21.51 | 5.22/5.18 | 12 | 66% | 149 | 1.30E-09 | 3.32±0.22 |
| **Function unknown** | | | | | | | | | | |
| 216 | ZP_06380822 | ORF1456 | Pentapeptide repeat-containing protein [*Arthrospira platensis*] | 19.86/24.68 | 5.13/5.44 | 16 | 58% | 125 | 3.30E-07 | 3.32±0.29 |
| 151 | ZP_03274838 | / | Conserved hypothetical protein [*Arthrospira maxima*] | 18.68/19.87 | 5.33/5.17 | 11 | 49% | 117 | 2.10E-06 | 3.06±0.13 |
| 158 | ZP_06383329 | ORF4728 | Hypothetical protein aplap_16770 [*Arthrospira platensis*] | 19.34/21.31 | 5.30/5.83 | 9 | 59% | 120 | 1.00E-06 | + |
| 68 | ZP_06384870 | ORF4030 | Hypothetical protein aplap_24737 [*Arthrospira platensis*] | 17.32/15.87 | 5.96/6.23 | 20 | 93% | 224 | 4.20E-17 | 3.19±0.21 |
| 65 | ZP_06384870 | ORF4030 | Hypothetical protein aplap_24737 [*Arthrospira platensis*] | 17.32/15.87 | 5.96/6.23 | 18 | 86% | 245 | 3.30E-19 | 5.57±0.42 |
| 78 | ZP_06384870 | ORF4030 | Hypothetical protein aplap_24737 [*Arthrospira platensis*] | 17.32/16.31 | 5.96/6.14 | 19 | 86% | 254 | 4.10E-20 | - |
| 98 | ZP_06384870 | ORF4030 | Hypothetical protein aplap_24737 [*Arthrospira platensis*] | 17.32/17.19 | 5.96/6.50 | 18 | 82% | 214 | 4.10E-16 | - |
| 339 | ZP_06384062 | ORF0361 | Peptidase S8 and S53 subtilisin kexin sedolisin  [*Arthrospira platensis*] | 44.24/31.22 | 4.59/4.65 | 16 | 44% | 200 | 1.00E-14 | - |

**Table S3C**

| **Spot#** | **Accession NO.** | **ORF** | **Gene product** | **Theor./** | **Exper.** | **Matched Peptides** | **Cov** | **Score** | **E-value** | **Fold change**  **(Mean± SD**) |
| --- | --- | --- | --- | --- | --- | --- | --- | --- | --- | --- |
|  |  |  |  | **MW(kDa)** | **pI** |  |  |  |  |  |
| **Amino acid transport and metabolism** | | | | | | | | | | |
| 516 | ZP_06381532 | ORF1242 | Cysteine synthase [*Arthrospira platensis*] | 34.53/33.33 | 5.92/6.38 | 19 | 44% | 150 | 1.00E-09 | +^45^ |
| **Carbohydrate transport and metabolism** | | | |  |  |  |  |  |  |  |
| 987 | ZP_06382051 | ORF4691 | Transketolase [*Arthrospira platensis*] | 72.98/77.27 | 5.78/5.53 | 16 | 29% | 104 | 4.20E-05 | +^15^ |
| 808 | ZP_03275023 | ORF2124 | Carbohydrate-selective porin oprb [*Arthrospira maxima*] | 41.00/49.88 | 7.96/6.39 | 20 | 32% | 99 | 1.30E-04 | +5.60^15^  ±0.32 |
| 986 | ZP_06382051 | ORF4691 | Transketolase [*Arthrospira platensis*] | 72.98/75.45 | 5.78/5.62 | 34 | 60% | 216 | 2.60E-16 | +3.45^15^  ±0.28 |
| **Coenzyme metabolism** | | | |  |  |  |  |  |  |  |
| 669 | ZP_03272326 | ORF5578 | UBA/THIF-type NAD/FAD binding protein [*Arthrospira maxima*] | 43.01/42.01 | 5.06/4.99 | 15 | 50% | 125 | 3.30E-07 | +^15^ |
| 256 | ZP_05256482 | ORF1829 | Radical SAM domain-containing protein [*Bacteroides*] | 36.20/28.14 | 6.21/6.59 | 10 | 35% | 77 | 2.10E-02 | +^15^ |
| **Post-translational modification, protein turnover, chaperones** | | | | | |  |  |  |  |  |
| 238 | ZP_06380315 | ORF0279 | Alkyl hydroperoxide reductase/ Thiol specific antioxidant/ Mal allergen [*Arthrospira platensis*] | 24.01/27.31 | 4.89/5.07 | 11 | 35% | 140 | 1.00E-08 | +3.74^15^  ±0.36 |
| **Translation, ribosomal structure and biogenesis** | | | | |  |  |  |  |  |  |
| 859 | ZP_03275668 | ORF4248 | Cysteinyl-trna synthetase [*Arthrospira maxima*] | 55.01/53.51 | 5.87/6.50 | 19 | 38% | 76 | 2.80E-02 | +^15^ |
| 514 | ZP_06380559 | ORF1074 | Elongation factor TS [*Arthrospira platensis*] | 24.63/33.40 | 5.74/5.90 | 18 | 70% | 148 | 1.70E-09 | +^45^ |
| **Energy metabolism (photosynthesis, respiratory electron transport)** | | | | | |  |  |  |  |  |
| 332 | ZP_03273564 | ORF2382 | Ferredoxin [*Arthrospira maxima*] | 27.02/31.29 | 5.68/5.78 | 14 | 47% | 101 | 8.30E-05 | +^15^ |
| 332* | ZP_06380716 | ORF2382 | Bidirectional hydrogenase complex protein hoxu [*Arthrospira platensis*] | 27.00/31.29 | 5.54/5.78 | 17 | 68% | 143 | 5.20E-09 | +^15^ |
| 436 | ZP_06380688 | ORF4632 | Phycobilisome linker polypeptide [*Arthrospira platensis*] | 32.78/34.72 | 8.33/5.38 | 18 | 48% | 129 | 1.30E-07 | +3.48^15^  ±0.31 |
| 478 | ZP_06382427 | ORF2155 | Phycobilisome linker polypeptide [*Arthrospira platensis*] | 29.45/32.15 | 9.25/5.85 | 25 | 70% | 206 | 2.60E-15 | +^45^ |
| **Nucleotide transport and metabolism** | | | |  |  |  |  |  |  |  |
| 877 | ZP_06382529 | ORF3386 | Dihydroorotase [*Arthrospira platensis*] | 48.64/55.32 | 5.45/5.86 | 15 | 27% | 102 | 6.60E-05 | +3.42^15^  ±0.34 |
| **Cell envelope biogenesis, outer membrane** | | | |  |  |  |  |  |  |  |
| 772 | ZP_06381274 | ORF0324 | UDP-glucose/GDP-mannose dehydrogenase [*Arthrospira platensis*] | 49.93/47.00 | 5.18/5.14 | 19 | 47% | 110 | 1.00E-05 | +^15^ |
| 941 | ZP_03275087 | ORF0324 | Nucleotide sugar dehydrogenase [*Arthrospira maxima*] | 34.72/47.54 | 5.26/5.16 | 30 | 72% | 249 | 1.30E-19 | +^45^ |
| **Function unknown** | | | | | | | | | | |
| 118 | ZP_06382587 |  | Hypothetical protein aplap_12998 [*Arthrospira platensis*] | 19.30/17.10 | 9.47/6.55 | 15 | 74% | 183 | 5.20E-13 | +^15^ |
| 183 | ZP_03274017 | ORF1894 | Uncharacterized protein/domain associated with gtpase-like protein [*Arthrospira maxima*] | 20.09/23.37 | 5.74/5.93 | 5 | 32% | 78 | 1.80E-02 | +^45^ |
| 425 | ZP_06384062 | ORF0361 | Peptidase S8 and S53 subtilisin kexin sedolisin [*Arthrospira platensis*] | 44.24/30.81 | 4.59/4.46 | 24 | 54% | 156 | 2.60E-10 | +^45^ |
